# Antiferromagnetic magnon spintronic based on nonreciprocal and nondegenerated ultra-fast spin-waves in the canted antiferromagnet α-Fe_2_O_3_

**DOI:** 10.1126/sciadv.adh1601

**Published:** 2023-08-11

**Authors:** Aya El Kanj, Olena Gomonay, Isabella Boventer, Paolo Bortolotti, Vincent Cros, Abdelmadjid Anane, Romain Lebrun

**Affiliations:** ^1^ Unité Mixte de Physique, CNRS, Thales, Université Paris-Saclay, 91767 Palaiseau, France.; ^2^ Institute of Physics, Johannes Gutenberg-University Mainz, 55128 Mainz, Germany.

## Abstract

Spin-waves in antiferromagnets hold the prospects for the development of faster, less power-hungry electronics and promising physics based on spin superfluids and coherent magnon condensates. For both these perspectives, addressing electrically coherent antiferromagnetic spin-waves is of importance, a prerequisite that has been so far elusive, because, unlike ferromagnets, antiferromagnets couple weakly to radiofrequency fields. Here, we demonstrate the detection of ultra-fast nonreciprocal spin-waves in the dipolar exchange regime of a canted antiferromagnet using both inductive and spintronic transducers. Using time-of-flight spin-wave spectroscopy on hematite (α-Fe_2_O_3_), we find that the magnon wave packets can propagate as fast as 20 kilometers/second for reciprocal bulk spin-wave modes and up to 6 kilometers/second for surface spin-waves propagating parallel to the antiferromagnetic Néel vector. We lastly achieve efficient electrical detection of nonreciprocal spin-wave transport using nonlocal inverse spin-Hall effects. The electrical detection of coherent nonreciprocal antiferromagnetic spin-waves paves the way for the development of antiferromagnetic and altermagnet-based magnonic devices.

## Teaser

Dipole-exchange antiferromagnetic spin-waves host the potential for non-reciprocal and ultra-fast magnonic devices.

## INTRODUCTION

Spin-wave dynamics in antiferromagnets hold the prospect of magnonic devices operating at the sub-terahertz frequencies (*1*–*3*) with a large group velocity (>10 km/s) by benefiting from their strong exchange field and quadratic spin-wave dispersion (*4*, *5*). In this context, antiferromagnetic spin-waves in the long and short (including dipole exchange modes) wavelength limits have been extensively investigated theoretically already some decades ago (*6*–*9*). For magnonic devices, one of the most basic actions to be realized is to be able to electrically excite and detect the corresponding fast spin-waves. However, up to now, there are no experimental observations of propagating properties of spin-waves in antiferromagnets (AFMs), in both direct and reciprocal space. Contrary to their counterparts, ferromagnets, in which large stray fields allow the inductive detection of the spin-wave dynamics relatively straightforwardly, these dipolar fields in antiferromagnets are zero or largely negligible. Beyond their key role in spin-wave detection, the non-compensated dipolar fields also provide some of the unique features such as nonreciprocity, magnetostatic spin-waves (*10*, *11*), and Bose-Einstein condensation (*12*, *13*) of conventional ferromagnet-based magnonic devices. Because of the bulk Dzyaloshinskii-Moriya interaction (*14*, *15*), canted antiferromagnets are anticipated to present more pronounced dipole exchange spin-wave modes in the small wave vector **k** region (< 6 rad/μm) (*16*–*19*) as required to facilitate their observations using standard inductive detection. Recently, many of these canted antiferromagnet materials, such as hematite and orthoferrites, have also been identified as altermagnets (*20*, *21*), a class of magnetic materials with opposite spin sublattices and a nearly vanishing compensated magnetic order but at the same time a broken T-symmetry leading to spin splitting in the momentum space. Such a lifted degeneracy of the electronic spin and magnon band structures shall enable to open to antiferromagnets, the same rich physics of spin current transport and spin-wave dynamics (*22*) than in ferromagnets (*23*). In this sense, insulating canted antiferromagnets such as hematite, the material to be studied here, or orthoferrites—with resonance frequencies ranging from 10 to 600 GHz (*24*–*26*), Dzyaloshinskii-Moriya interaction (DMI) fields from 1 to 20 T (*27*), and low magnetic damping (*25*, *28*, *29*)—are thus prime candidates to develop the field of antiferromagnetic and alter-magnonics. In the past decade, research in spintronic proposed various approaches, enabling the detection and the manipulation of antiferromagnetic spin-waves using the spin-to-charge phenomena (*30*–*32*). Until now, electrical detection based on the inverse spin-Hall effect was achieved only for the uniform mode (**k** = 0) with generated voltage amplitudes as low as tens of nanovolt in both colinear (*2*, *3*) and canted antiferromagnets (*33*, *34*).

In this article, we successfully identify magnetostatic spin-waves for low **k** vector (0.1 to 2.3 rad/μm) in hematite (α-Fe_2_O_3_). To this aim, we first used spin-wave spectroscopy between two inductive transducer antennas, allowing us to detect these AFM spin-waves after propagating on a distance of more than 10 μm. Using time-of-flight spin-wave spectroscopy (*35*), we evidence the presence of different spin-wave packets with very large group velocities ranging from 5 to 30 km/s. In addition, we report a strongly lifted degeneracy of the bulk spin-wave band for **k** perpendicular (⟂) or parallel (//) to the antiferromagnetic order **n**, with a separation larger than 1 GHz at k = 0.6 rad/μm and demonstrate the nonreciprocal character of spin-wave modes for **k** // **n**, a highly interesting feature for the development of antiferromagnetic magnonics. Last, we achieve electrical detection of the nonreciprocal antiferromagnetic spin-waves with a platinum-based metallic transducer (through the inverse spin-Hall effect) with microvolt output voltages, as in ferromagnets such as Ytrium-Iron Garnet (YIG) (*36*, *37*). Our observations evidence that spintronic transducers represent a promising alternative to detect antiferromagnetic spin-waves with reduced dipolar fields.

## RESULTS

### Lifting of magnon degeneracy in canted antiferromagnets

**Fig. 1.**
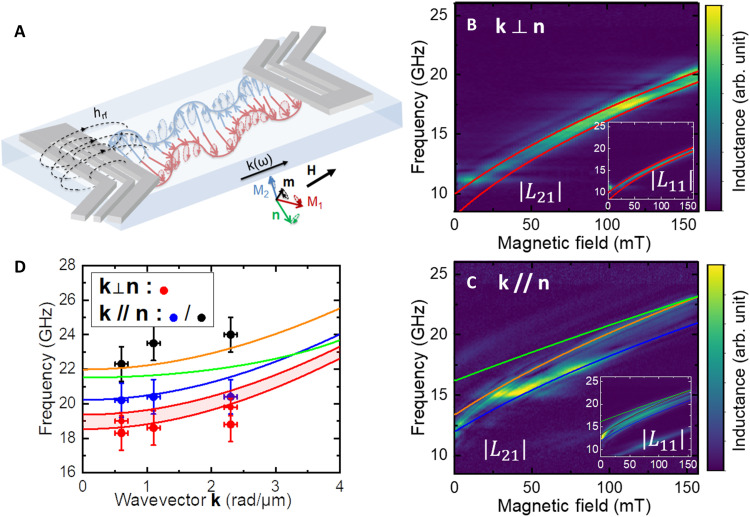
Spin-wave transport in the canted antiferromagnet α-Fe_2_O_3_. (**A**) Schematic of the setup. The net sublattice magnetization M_1_ and M_2_ have strongly elliptical trajectory, oscillating mainly in the sample plane (easy plane) with only a small opening angle in the out-of-plane direction. **n** and **m**, respectively, correspond to the Néel vector and the canted moment dynamics. **n** is linearly polarized in the sample plane, while **m** is elliptically polarized. The static net moment **m** is aligned along the applied field **H**, and the Néel vector **n** is perpendicular to it. (**B** and **C**) Spin-wave transmission measurement showing the transmitted amplitude |*L*_21_| for (B) **k** ⟂ **n** and **k** // **n** for (C) at k ≈ 0.6 rad/μm. Red and blue lines correspond to fits using the theoretical bulk spin-wave equations for **k** // **n** and **k** ⟂ **n**, respectively. Orange and green lines, respectively, correspond to a modeling of the high-frequency spin-wave branch for **k** // **n**, assuming a bulk or a surface mode [see section S7 and (*16*, *17*, *19*)]. Insets show the amplitude of the reflected signal |*L*_11_|. (**D**) Magnon branch dispersion for **k** // **n** and **k** ⟂ **n** at a magnetic field of 140 mT. (Blue and red lines correspond to the theoretical bulk spin-wave branches **k** // **n** and **k** ⟂ **n**, respectively, using the fitting in magnetic field).

We excite and detect propagating spin-waves in c-plane-oriented single crystals of the canted antiferromagnet α-Fe_2_O_3_ (*14*, *38*, *39*) by means of propagative spin-wave spectroscopy (*40*) (cf. Fig. 1A). First, we measure with a vector network analyzer (VNA) the reflected *L*_11_ and transmission *L*_21_ parameters between two inductive antennas that predominantly excite spin-wave with **k** vector of 0.6 rad/μm (see more details in Materials and Methods and section S1). We succeed in detecting spin-wave propagation for an edge to edge distances as large as 14 μm. Such a long-distance coherent transport of antiferromagnetic spin-waves is in line with the recently reported micrometer-long magnon spin diffusion length and the ultra-low magnetic damping of hematite (*28*). Because of the small canted moment **m** of α-Fe_2_O_3_ [M_s_ ≈ 3 emu/cm^3^ (*38*)], the direction of the antiferromagnetic order **n** can be oriented perpendicular to the applied field **H** with fields as low as 50 mT (*38*). Hematite is one of the rare easy-plane antiferromagnet at room temperature, and we specifically chose the sample to have the magnetic easy-plane parallel to the surface. This property allows us to investigate spin-wave propagation for an antiferromagnetic order **n**, either parallel or perpendicular to the spin-wave vector **k**. As described hereafter, these measurements lead to the observation of notable different behaviors for **k** // **n** and **k** ⟂ **n**. First, for **k** ⟂ **n** (i.e., **H** // **k**), we observe, as shown in Fig. 1B, two close spin-wave branches, around 19 GHz at 150 mT. This could be associated to slightly nonuniform anisotropies of the sample (*28*, *41*). For **k** // **n** (i.e., **H** ⟂ **k**), we observe a main spin-wave mode (blue line), as shown in Fig. 1C, along with several spin-wave branches at slightly higher frequencies (represented by orange and green lines). These features could indicate the presence of magnetostatic modes (*16*, *19*, *28*, *42*), and we will discuss later how to identify them. It is to be noticed that the lowest spin-wave branch (blue branch) for **k** // **n** follows a similar frequency dispersion as for **k** ⟂ **n** but is always higher in frequency by about 1 GHz. We also see that the signal amplitude strongly varies below 50 mT, this is due to, as mentioned above, the reorientation of the Néel vector **n** and canted moment **m** (*28*, *43*) in this low field range.

To understand the origin of this anisotropic magnon transport, we measure in Fig. 1D the spin-wave dispersion for **k** // **n** (black and blue points) and **k** ⟂ **n** (red points) by performing spin-wave spectroscopy at different **k** vectors using several transducer designs (see section S1). We observe the persistence of well-separated magnon branches for the two configurations, with always higher frequencies for **k** // **n** (see section S3). Such a lifted degeneracy of the magnon dispersion is not expected from the standard degenerated linear dispersion reported for antiferromagnets. To go beyond, other regimes should be considered, such as the dipole exchange regime of canted antiferromagnets, which, in our knowledge, has not been yet explored experimentally. As for the difference in spin-wave frequency between the two configurations **k** ⟂ **n** and **k** // **n**, some theoretical models (*16*, *17*) do predict that the bulk spin-wave dispersion should vary between these two cases. The refined expression of the bulk spin-wave bands (see section S5) in the dipole exchange regime leads to a frequency difference $\text{Δ}f_{\mathrm{SW}}=f_{k//n}-f_{k⊥n}=\sqrt{f_{10}^{2}+\frac{4\pi M_{s}}{H_{\mathrm{ex}}}{\left(\frac{\gamma }{2\pi }\right)}^{2}{\left(H+H_{\mathrm{DMI}}\right)}^{2}}-f_{10}\left(1+\frac{4\pi M_{s}}{H_{\mathrm{ex}}}\right)$ with *f*_10_ as the frequency gap for the lowest magnon mode (*33*, *41*), γ is the gyromagnetic ratio, *H*_ex_ is the exchange field, and *H*_DMI_ is the Dzyaloshinskii-Moriya field. Using the material parameters of hematite (see Materials and Methods), we estimate ∆*f*_SW_ ≈ 0.5 to 1 GHz for small **k** vectors (10 rad/μm), which agrees with the observation that the spin-wave frequencies are higher for **k** // **n** than for **k** ⟂ **n**. We can fit the frequency of the bulk spin-wave modes versus fields for these two configurations (see, respectively, red and blue in Fig. 1, B and C). This result highlights the importance of magnetostatic interactions in the spin-wave dynamics of canted antiferromagnets at small **k** vectors (<10 rad/μm). However, these models cannot explain the presence of the higher frequency spin-wave branches present for **k** // **n**.

### Time of flight of surface and bulk antiferromagnetic spin-waves

**Fig. 2.**
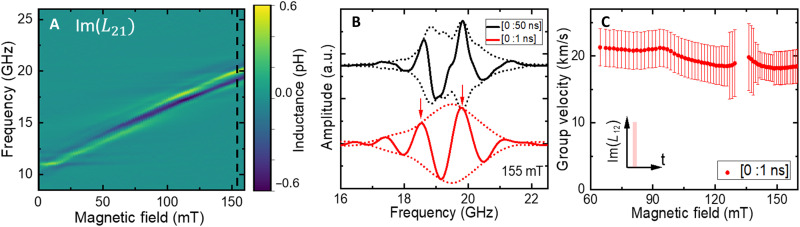
Ultra-fast antiferromagnetic spin-waves for k ⟂ n revealed by time-gated VNA measurements. (**A**) Imaginary part of the transmitted spin-wave Im(*L*_21_) as a function of field without time gating. (**B**) Example of Im(*L*_21_) spectra of the full spin-wave signals (time gate of [0:50 ns], black) and of the main spin-wave packet (time gate of [0:1 ns], red) for H = 155 mT (no spin-wave signal is detected after 10 ns). For an edge-to-edge antenna distance of 14 μm, oscillations (red) indicate a spin-wave group velocity of >14 km/s. Dotted lines correspond to the signal envelopes. (**C**) Group velocity of the main spin-wave packet for a time gating of [0:1 ns]. Error bars are defined as the noise level from the imaginary part of the transmitted inductance *L*_21_. a.u., arbitrary units.

To get more insights about the properties of these propagating AFM spin-waves, we analyze their amplitude and their phase in more details for **k** // **n** and **k** ⟂ **n**. We restrict our analysis for magnetic fields above 50 mT to ensure that the Néel vector **n** is always strictly perpendicular to **H**. In Fig. 2A, we present the imaginary part of the transmitted spin-wave spectra *L*_21_ for **k** ⟂ **n**. As shown in Fig. 2B, we observe the expected oscillatory behavior of the phase delay ϕ = *kD*_ant_ (with *D*_ant_ as the distance between the two transducer antennae) accumulated by the spin-wave during its propagation. From these oscillations, the spin-wave group velocity $v_{g}=\frac{\partial f}{\partial k}∼D_{\mathrm{ant}}\text{Δ}f$ can be extracted from the periodicity of phase oscillations Δ*f*. However, as shown in the black curve of Fig. 2B, the envelope of the signal shows the presence of more than one spin-wave packet. Those are due to both the wide k bandwidth of our antenna (*∂k* ≈ 0.2 to 1 rad/μm; see section S1) and potentially to propagating spin-waves with a nonuniform thickness profile in our 500-μm-thick film. To access the group velocity of each spin-wave packet, we perform time-gating VNA measurements (*35*) with different time intervals (see section S2). As shown in Fig. 2B, we detect the main (and fastest) spin-wave packets in less than 1 ns of traveling time for an edge-to-edge distance of 14 μm between the antennas, indicating a minimum group velocity of >14 km/s. Note that a traveling time of 1 ns (no spin-wave packet after 2 ns) is compatible with the group velocity that can be extracted from the phase oscillations Δ*f* in Fig. 2C, which lies in an average value of around 20 km/s over the measured field range. We emphasize that a group velocity as our reported value represents a record velocity for spin-waves in a magnonic device.

Contrary to ferromagnets in which the group velocity at small **k** scales with the magnetization saturation *M*_s_ (*44*), the group velocity *v*_g_ in both collinear and canted antiferromagnets is proportional to *H*_ex_ (*45*). Thus, in antiferromagnets, it results that the group velocity can reach tens of kilometers per second as observed in the present work (*4*) or that domain wall velocity can be a few kilometers per second as in orthoferrites (*5*). Note that the observed large spin-wave velocity is also in agreement with the value estimated from the experimental slope of the spin-wave dispersion $\frac{\partial f}{\partial k}$ presented in Fig. 1D, which also corresponds to spin-wave velocity larger than 10 km/s.

**Fig. 3.**
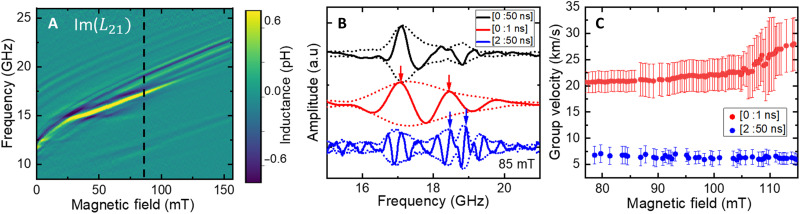
Ultra-fast antiferromagnetic spin-waves for k // n. (**A**) Imaginary part of the transmitted spin-wave Im(*L*_21_). (**B**) Exemplary spectra of Im(*L*_21_) for H = 85 mT for different time gating, [0:50 ns]: full spin-wave signals (black), [0:1 ns]: first spin-wave packet (red), [2:50 ns]: secondary spin-wave packets (blue). Dotted lines correspond to the signal envelopes. (**C**) Group velocity of the different spin-wave packets. The antenna shows a **k** selectivity centered around 0.6 rad/μm (see the design in Materials and Methods). Error bars are defined as the noise level from the imaginary part of the transmitted inductance *L*_21_. Higher frequency modes propagate around three times slower with around 8 km/s than the bulk mode, with about 20 km/s.

In Fig. 3, we present the spin-wave propagating properties in the geometry **k** // **n**. As mentioned before, spin-wave branches separated by a few gigahertz can be observed in this case. While the first one can be associated to bulk spin-wave, the higher frequency ones could correspond to the predicted surface spin-wave modes or hybrid surface-bulk modes (see section S5) (*19*, *41*, *42*, *46*). In Fig. 3, we thus present time-gating measurements to independently access these different spin-waves modes. As for the configuration **k** ⟂ **n**, we observe that the first (and fastest) spin-wave packet travels in less than 1 ns and exhibits a group velocity of about 20 km/s (see Fig. 3C). It slightly increases with the field, leading to larger phase oscillations Δ*f* that become difficult to extract above 120 mT. Unexpectedly, the higher frequency spin-wave modes (see blue curves in Fig. 3, B and C) propagate more slowly but still travel in less than 10 ns. As they are close in frequencies and have similar traveling times, we only determine the average group velocities to be around 6 km/s.

### Nonreciprocal spin-wave transport

**Fig. 4.**
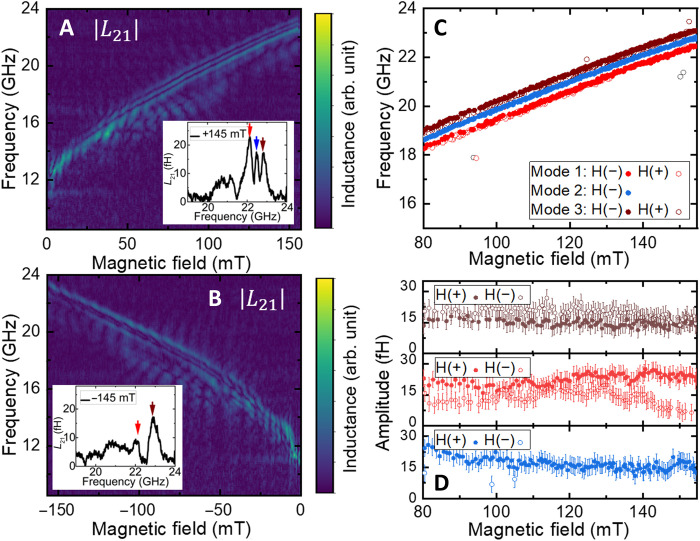
Nonreciprocal spin-wave for k // n. (**A** and **B**) Absolute value of the transmitted spin-wave spectra ∣*L*_21_∣ with a time gating of [2:50 ns] for positive fields in (A) and negative in (B). Insets show exemplary spectra for H, respectively, of +145 and −145 mT. Arrows indicate the position of the three different modes. (**C**) Frequency and (**D**) amplitude of the three main spin-wave modes for negative and positive fields. Error bars are defined as the noise level from the transmitted inductance *L*_21_.

Nonreciprocity is a key property for many spin-wave analog devices (such as circulators). It has been widely studied in ferromagnets in the presence of surface spin-wave modes (*47*) but, up to our knowledge, only predicted in antiferromagnets (*7*, *8*). Here, we thus investigate the potential nonreciprocity of the high-frequency spin-wave packets for **k** // **n**. In Fig. 4 (A and B), we present the amplitude of the transmitted spin-wave packets ∣*L*_21_∣ for positive and negative fields, respectively. We use a time gating of [2:50 ns] to select the high-frequency spin-wave modes (see Fig. 3A). As seen in Fig. 4C, we do not observe a sizeable frequency shift between positive and negative magnetic fields. However, as far as the spin-wave amplitude is concerned, we find a clear nonreciprocity for two of the three spin-wave modes. As shown in Fig. 4D, we observe for negative magnetic fields a reduction by about a factor 2 of the red mode and even the absence of the blue mode. This nonreciprocal behavior is confirmed by measuring different amplitudes for ∣*L*_21_∣ and ∣*L*_12_∣ parameters (see section S6). These results are signatures of surface spin-wave modes propagating with opposite directions at the two surfaces of the sample for **k** // **n**, which are expected also in case of an antiferromagnet (*7*, *8*, *19*). On the contrary, for **k** ⟂ **n,** we measure similar spin-wave amplitudes for the different spin-wave packets between positive and negative fields and between *L*_12_ and *L*_21_ parameters (see section S5), indicating a reciprocal behavior in this configuration (*7*, *8*). To understand, in more details, the symmetry of these spin-waves and how they decay within the AFM requires a further theoretical investigation, but it remains beyond the scope of this work. This can be done by using either the canted antiferromagnet approach (see section S5) or the altermagnet formalism.

### Inverse spin-Hall detection of nonreciprocal spin-wave propagation

**Fig. 5.**
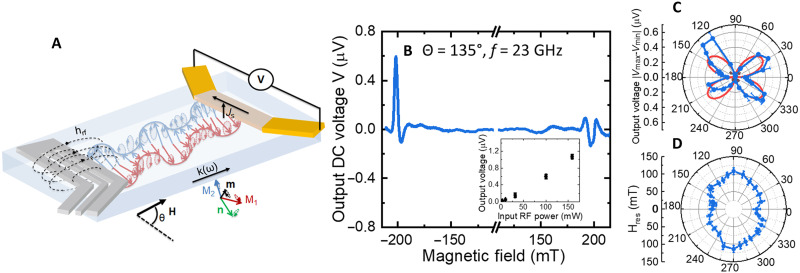
Detection of antiferromagnetic spin-wave by inverse spin-Hall effects. (**A**) Sketch of the devices. An inductive transducer as in Fig. 1 is used to excite the coherent antiferromagnetic spin-wave, and a platinum-based detector is used to detect the DC inverse spin-Hall voltage generated by the propagation spin-waves. (**B**) Example of inverse spin-Hall spectra for a magnetic field applied at θ = 135° from the inductive transducer (with an input power *P*_rf_ = +16 dBm). Inset: Power dependency of the peak-to-peak output voltage for *f* = 17 GHz and θ = 45°. (**C**) Angular dependency of the DC inverse spin-Hall voltage *V*_pp_ for a 14-μm distance between the injector and the detector for *f* = 17 GHz. The red line corresponds to the fit with a (*A* cos θ^2^ + *B*)∣ sin θ∣. (**D**) Angular dependency of the resonance field for *f* = 17 GHz. RF, radiofrequency.

A key challenge in magnonic devices is the amplitude of the output voltage generated by the propagating spin-waves, while efficient alternatives to standard inductive transducers (*48*) are still lacking. This challenge is even amplified in antiferromagnetic materials, given the reduced generated stray field. Here, we lastly achieve efficient electrical detection of the propagating spin-waves through the surface-sensitive inverse spin-Hall effect using a platinum-based metallic transducer (see sketch in Fig. 5A). As seen in Fig. 5B, we observe a sign reversal of the generated DC voltage for positive and negative fields, indicating its spin-pumping nature. Another important feature is the strong asymmetry (about 40%) of the output voltage, which indicates the nonreciprocity of the detected spin-waves in line with our previous observations and evidences their presence at the surface of the crystal. We also study the angular dependency of the inverse spin-Hall voltage in Fig. 5 (C and D). In Fig. 5D, we observe that the resonance field at 17 GHz is larger for **k** // **n** than for **k** ⟂ **n**, which confirms the results from Fig. 1. Furthermore, we notice in Fig. 5C that the detected output voltage follows an asymmetric (*A* cos θ^2^ + *B*) sin θ dependency, with maxima for external magnetic field applied at 45° and 135°, from the transducer direction. This feature is in accordance with an excitation efficiency of the inductive transducers, which follows an (*A* cos θ^2^ + *B*) law (see section S6), and inverse spin-Hall detection, which follows a standard sin θ law (*33*, *37*). The additional asymmetry arises from the spin-wave nonreciprocity discussed in the previous section. One should mention that the shape of the output voltage peak can change toward high power due to nonlinear effects coming into play (arising from the ultra-low damping of hematite) that would require further study. By comparing the voltage amplitude for two distances between the injector and the detector, we also extract an attenuation length of about 3 to 4 μm. As shown in the inset of Fig. 5B, we measure output inverse spin-Hall voltages in the microvolt range, while the excitation frequency is one order of magnitude larger than in ferromagnets (*36*, *37*). This further evidences that spin-pumping effects represent a promising tool to detect the spin-wave dynamics in antiferromagnets and favorize their integration in magnonic devices.

## DISCUSSION

We thus electrically detect by both inductive and spintronic transducers the presence of nondegenerated and nonreciprocal spin-waves in the dipolar exchange regime of a canted antiferromagnet, with record group velocities (of about 20 km/s) and micrometers propagation distances. We can well model the presence of a bulk spin-wave frequency band of a few gigahertz with a lifted degeneracy for **k** ⟂ **n** and **k** // **n**, which is anticipated to be a generic feature for canted antiferromagnets at low **k** vectors. Furthermore, for **k** // **n**, we observe the coexistence of nonreciprocal with reciprocal spin-wave modes in Fig. 4. This nonreciprocal behavior is even enhanced at larger **k** (see section S4). Without considering the coupling between surface and bulk modes, we can theoretically determine the frequency of the antiferromagnetic surface spin-wave modes to be $f_{\mathrm{sur}}=\frac{f_{10}^{2}+{\left(\frac{ck}{2\pi }\right)}^{2}}{\frac{\gamma }{\pi }(H+H_{\mathrm{DMI}})}+\frac{\gamma }{4\pi }\left(1+\frac{4\pi M_{s}}{H_{\mathrm{ex}}}\right)(H+H_{\mathrm{DMI}})$. Using the material parameters of hematite, we would thus expect spin-wave surface modes at around 32 GHz at 100 mT for **k** ≈ 0.6 rad/μm. This value is definitively larger than our experimental observations shown in Fig. 4, and furthermore, the required stability conditions to be localized on the surfaces are not fulfilled (See section S5). Hence, the frequency of stable surface modes at around 20 GHz can only be fitted with an unrealistic phenomenological effective DMI field (*H*_DMI_ ≈ 1.3 T; see green lines in Fig. 1B). However, similarly to what happens in thick ferromagnets (*46*), bulk and surface spin-waves can also strongly hybridize in a single crystal, leading to spin-wave modes with mixed properties. This could explain the presence of spin-wave modes close in frequency with either nonreciprocal or reciprocal spin-wave behaviors as observed here in Fig. 4. Overall, our findings can only partially be modeled with a standard theory of antiferromagnetic spin-waves (developed in more details in section S5). Thus, further theory works would require to investigate in more details the mode interaction and the hybridization depending on the system geometry (*16*, *17*, *19*), together with the altermagnetic character of α-Fe_2_O_3_ (*20*, *21*, *23*). The large spin-pumping signals generated by the propagating antiferromagnetic spin-waves also provide a promising tool to access their dynamics in both single crystals and thin films. One should notice that the large inverse spin-Hall voltage can be linked with the amplitude of the spin-Hall magnetoresistance (*39*, *49*) reported in bilayers of hematite/platinum, as large as in bilayers of YIG/platinum (*50*). Given the low magnetic damping of other few orthoferrites, the material class of canted antiferromagnets demonstrates all its potential for establishing a research field around antiferromagnetic and alter-magnonics, with a lot of opportunities for high-frequency magnonics.

Note that, in the preparation of this manuscript, we became aware of two recent works on spin-wave spectroscopy in hematite in which the authors also observed large group velocities of tens of kilometers per second and long propagation distances (*51*, *52*). Our work evidences that, due to the presence of the Dzyaloshinskii-Moriya field, the spin-wave dispersion in this canted antiferromagnet or altermagnet is nontrivial compared to the standard description of an antiferromagnet, being strongly nondegenerated due to magnetostatic interaction at small **k** vectors, and can show reciprocal and nonreciprocal behaviors. The propagating surface spin-wave can then be efficiently detected using spintronic transducers using inverse spin-Hall effects.

## MATERIALS AND METHODS

### Sample and nanofabrication procedure

The studied sample is a c-plane 500-μm-thick single crystal of the antiferromagnet α-Fe_2_O_3_ in its canted phase (at room temperature) (purchased from the company SurfaceNet). The magnetic properties of the crystal are similar to the ones detailed in (*2*–*3*).

For the devices (design 1, see section S1) with largest dimensions, optical lithography using SMART PRINT-UV has been used to pattern the ground-signal-ground (GSG) antenna on the sample, whereas for the smallest devices (designs 2 and 3), electron beam lithography has been used. Then, we deposit on top of the patterned structures a bilayer of 20 nm of Ti and 280 nm of Au by evaporation. Last, the sample is immersed in acetone to lift off the resist, thus keeping the metallic GSG antennae on top of the sample.

### Propagative spin-wave spectroscopy technique

In all our results, the S-matrix is transformed to an inductance matrix using the following equation

$$L(w)=\frac{Z_{0}}{jw}{\left[I_{2}-S(w)\right]}^{-1}[I_{2}+S(w)]$$

with $L(w)=\left(\begin{array}{cc} L_{11} & L_{12}\\ \;L_{21} & L_{22} \end{array}\right)$, $I_{2}=\left(\begin{array}{cc} 1 & 0\\ 0 & 1 \end{array}\right)$, and *Z*_0_ is the 50-ohm impedance of the VNA port.

#### Calibration of the VNA

Regardless of its high efficiency, VNA measurements assemble many losses from errors in the VNA internal circuit, its associated cabling, and the 40-GHz Z probes from FormFactor. Calibration is then needed to remove these losses. A Short-Open-Load-Thru (SOLT) calibration has been performed using a calibration substrate from FormFactor (with elements of known standards) and thus eliminates all parasitic signals until the pitch of the radiofrequency probes. Time 0 is defined as a direct short between the two pico probes. More explicitly in our case, the measurements are based on frequency domain with a time gate. The analysis primarily involves mathematical procedures. The measured signal in the frequency domain is transformed by an inverse Fourier transform (IFT) to the time domain, where a time gate [T_start_:T_end_] is applied. Only within this time window, the signal is analyzed, and then it can be converted back to the frequency domain using an FT.

#### Parameters of the measurements

In all the measurements, the VNA sweeps in the frequency domain, from 5 to 30 GHz with a step of 5 MHz (5001 points). The excitation power is fixed at −20 dBm to remain in the linear regime. The group velocity is calculated considering the edge-to-edge distance between the antennas.

## Supplementary Materials

### This PDF file includes:

Section S1 to S8 Figs. S1 to S18 Tables S1 and S2 References
