# Supplementary material for: Antiferromagnetic magnon spintronic based on nonreciprocal and nondegenerated ultra-fast spin-waves in the canted antiferromagnet α-Fe2O3
Source: Sci Adv. 2023 Aug 11;9(32):eadh1601. doi: 10.1126/sciadv.adh1601 (PMC10421035; doi:10.1126/sciadv.adh1601)
Supplement: Supplementary file 1 — Section S1 to S8 Figs. S1 to S18 Tables S1 and S2 References [file sciadv.adh1601_sm.pdf]

Science Advances
AAAS

Supplementary Materials for

Antiferromagnetic magnon spintronic based on nonreciprocal and nondegenerated ultra-fast spin-waves in the canted antiferromagnet α-Fe_2O_3

Aya El Kanj *et al.*

Corresponding author: Romain Lebrun, romain.lebrun@cnrs-thales.fr

Sci. Adv. **9**, eadh1601 (2023)
DOI: 10.1126/sciadv.adh1601

This PDF file includes:

Section S1 to S8
Figs. S1 to S18
Tables S1 and S2
References

Section 1. Antenna design and k-selectivity

For our study, we use radio-frequency antennas of ground-signal-ground (GSG) type separated by a distance *d*. When an rf-current is injected, the antenna generates a dynamic magnetic field that excite the SWs. Thus, the antennae's shape defines the wave vector of the excited SW. The antenna excitation spectrum is predicted from calculating the Fourier transform of the spatial current distribution in the antennas (*40*).

In this work, we have used only one geometry of antenna with 3 different ground-signal lines widths in order to select different k-vectors.

The widths and the k-values are represented in **Table S1**., where w_g and w_s are the widths for the ground and signal lines respectively. These two are separated by a distance *s*, the separation distance.

Device Name	w_g (µm)	w_s (µm)	s (µm)	k (rad/µm)
Design 1	2	4	2	0.6
Design 2	1	2	1	1.1
Design 3	0.5	1	0.5	2.3

Table S1: Antenna design. *Table showing the ground-signal-ground widths w_g and w_s and the separation distance s from a ground line to a signal line, for three devices, resulting in three different characteristic k-vector associated for each antenna.*

The current density profiles of the 3 different designs in k-space are shown in **Fig. S1**.

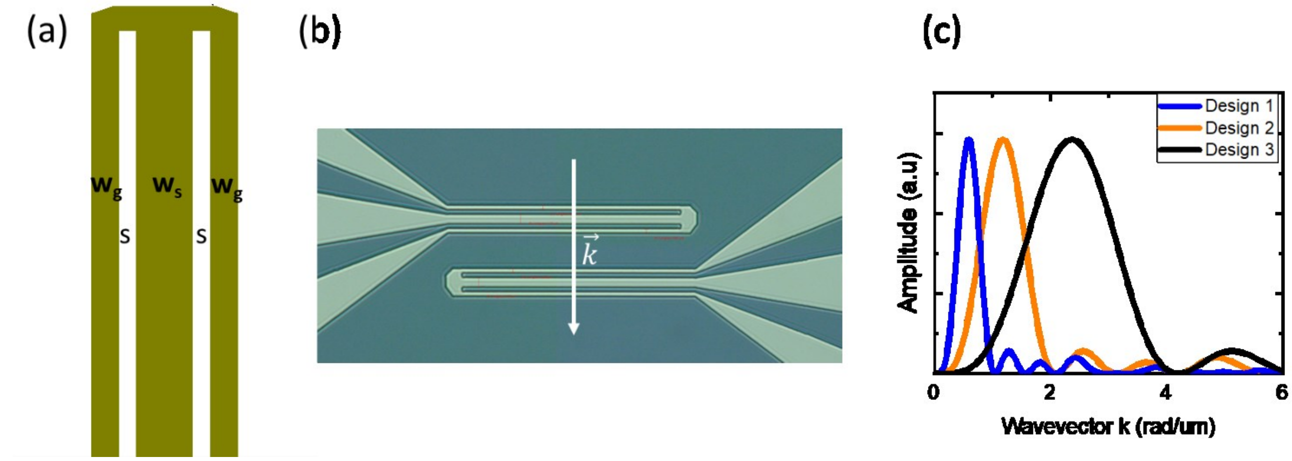

Figure S1: G-S-G antenna design*. (a) Schematic: the ground lines of width w_g are separated by a distance s from the central signal line of width w_s, (b) Optical microscope image for one design showing the direction of propagation of the spin-waves $\vec{k}$, (c) Current density profile of three different sizes of GSG antenna in function of the wavevector k and showing the maximum k-value i.e. the value at which the spin-wave is excited ; Each curve corresponds to one antenna in one design ; Blue for design 1 , orange for design 2 and black for design 3.*

Section 2. Spin-wave spectroscopy gating

a. Principle of time gating

The vector network analyzer permits measuring the scattering parameters (amplitude and phase) in the frequency domain. However, measuring in the time domain is an option available in most vector network analyzers(53) such as the ZVA 67 used in this study. This type of measurement is done through the inverse Fourier transform of the S-parameters in the frequency domain. Thus, this technique allows localizing the time at which the response hosts noise, or different signals propagating at different speeds. As a result, we can determine a 'gate' in time to measure a clean signal. Gating is a filter in the time domain, where the gate window is selected by fixing a starting and a stop gating times [T_{start}:T_{stop}], then the s-parameters are measured within this time window and are then transformed back to the frequency domain as shown in **Fig. S2**.

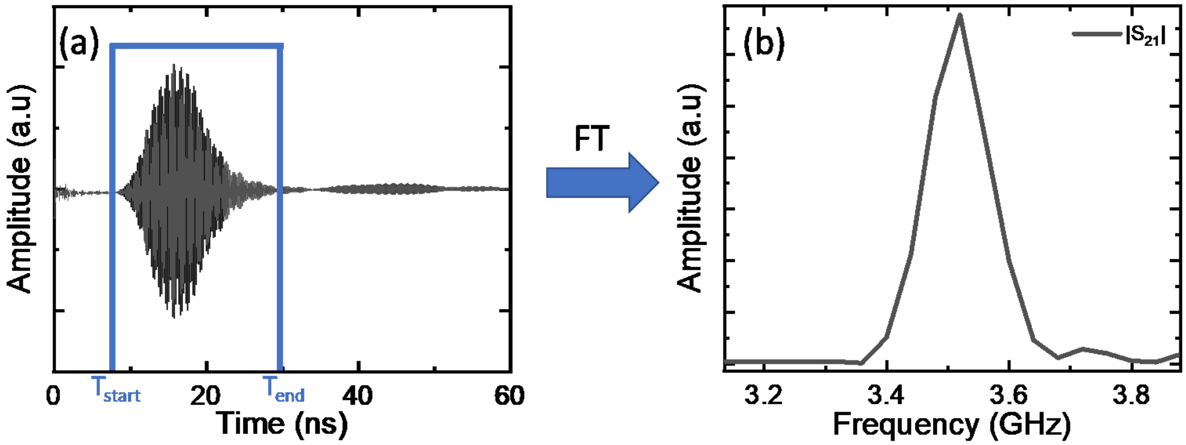

Figure S2: Principle of time domain spectroscopy: *(a) Exemplary spectra taken for a YIG film of 500 nm thickness, to enhance the signal amplitude in this example, for H = 100 mT, with an applied gate of boundaries T_{start}=8 ns and T_{end} =30 ns. (b) Fourier transform of the spectra in (a) showing a resonance frequency at 3.5 GHz.*

b. Frequency Domain with a Time Gate:

Different types of time domain measurements exist with a VNA [4]. Here we use the mode "Frequency with time gate", i.e. a standard frequency domain measurement, of which the data is then transformed mathematically to time domain where a gate (of a chosen shape) is applied. Thus, the data outside of the selected gate are removed before being converting back to the frequency domain. The data is processed by the analyser by using either a band pass mode or low pass mode. In the measurements shown in **Figure 4** in the main text, we have used "Frequency with Time gate", with a bandpass transformation. The difference between both modes is that in bandpass mode, the time zero-frequency point is not considered and the information on the phase is lost. Yet this mode is more convenient to use here since it does not include any restrictions on the frequency sweep points as it works for any set of equidistant points.

c. Time gating of spin-waves:

Spin waves possess rich dispersion relations that can be studied by various means, such as Brillouin light scattering (BLS) and inductive means (VNAs). It is possible that the material under study hosts several SWs branches (families) with different frequencies, yet at a very close frequency, one from the other, where the main mode is the most intense branch of the spectrum. This leads to a complex SW spectrum where the oscillations profile of the imaginary part of the transmission parameter presents either ripples or a mixture of small peaks contributing strongly to the main

response of the main branch, which complicates the calculation of the associated group velocity for each mode (*35*). Since each of these modes has a different group velocity, i.e., different delays, a way to differentiate between them is to use the time gating technique.

We performed measurements with different gates for the two experimental cases reported in the main text, either **k** ⊥ **n** (i.e. **H** // **k**), and **k** // **n** (i.e. **H** ⊥ **k**). For each configuration, we show the results obtained with the following cases of gates: No gate [0:50ns], gate [0:1 ns] and gate [2-50 ns].

In the case of α-Fe_2O_3 for k ⊥ n: Figure S3. (a-c) shows a color map of the imaginary part of the transmission inductance parameter L_{21} as a function of the swept frequencies and fields for three different time gatings.

1. Measurement with no gate [0:50ns]: In this measurement shown in **Fig. S3 (a)**, we perform a regular frequency domain measurement without gating the signal as no detectable spin-wave signal propagates with a time travel larger than 20 ns in our system. When no gate is applied, we observe clear oscillations of the transmitted inductance with two main intense spin-wave maxima separated by a Δf of about 2 GHz. However, as mentioned in the main text, within this gap of 2 GHz, we observe weaker peaks, yet these modes are hindered by the main ones.
2. Measurement with Gate [0:1ns]: In this measurement we perform a frequency domain measurement with gating. We apply a gate that starts from 0 ns and cuts the signal after 1 ns. We observe cleared oscillations of the inductance L_{21} as shown in **Fig. S3 (b)**. We can then fit field-by-field the position in frequency of these peaks, and we can extract the group velocity of the associated spin-wave mode from the evolution of Δf as shown in **Fig. 2 (c)** of the main text.

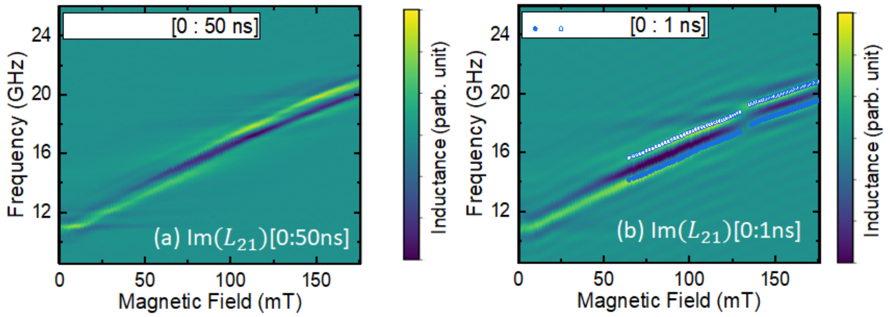

Figure S3: Imaginary part of the transmission spectra L_{21} at different gates for k ⊥ n: *(a) The case without applying a time gate (i.e. measuring from 0 ns to 50ns). (b) Case of a gate starting at 0 ns to 1 ns , the blue lines are fitted peaks of the imaginary part of L_{21}.*

In the case of α-Fe_2O_3 for k // n: Figure S4. (a-c) displays a color map of the imaginary part of the transmission inductance parameter L_{21} in function of the swept frequencies and fields for three different time gatings.

1. Measurement with no gate [0:50ns]: With no gate applied, the imaginary part of the L_{21} parameter in **Fig. S4. a** shows a richer spectrum in peak intensities than in the previous case (**H** // **k**). The fits of the peaks used to calculate the spin-wave group velocity are presented as black dots.
2. Measurement with a gate [0:1ns]: Due to the complex spectrum, the use of gating is necessary to differentiate between the different peaks. By gating from 0 ns to 1 ns we attempt to isolate the fastest propagating mode. By doing so we cut from the measured the signal the contribution of the slower modes and also all other measurement losses. From the fits shown in **Fig. S4 (b)** (blue lines) we were able to extract the group velocity by following

three neighbouring peaks i.e. two modes and we find group velocities of higher values than in the case without gating.

3. Measurement with Gate [2:50ns]: Now, we do a gate starting from 2 to 50 ns in order to isolate the slower mode. We observe that the *Δf* between neighbouring branches has indeed shrinked resulting in lower group velocity (see **Fig. 3** in the main text).

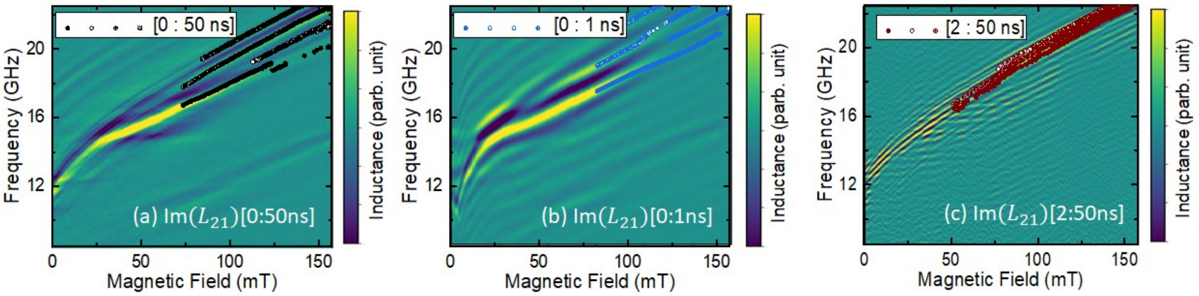

Figure S4: Imaginary part of the transmission spectra L_{21} at different gates for k // n: *(a) The case without applying a time gate (i.e. measuring from 0ns to 50ns), the black straight lines are the fitted peaks of the imaginary of L_{21}. (b) Case of a gate starting at 0ns to 1 ns , the blue lines are the fitted peaks of the imaginary part of L_{21}, (c) Case of a gate starting from 2 ns to 50 ns, the red lines are the fitted peaks of im(L_{21})*

Section 3. Spin-wave spectroscopy for k at different k-values.

In this section, we show the results of the measurements done on designs 2 and 3 (**Table S1**). We present here the measurements done for both directions of **k**, with the exact parameters of design 1 (**Section S4**) and with no gating applied.

We show in **Figure S5 (a-c)** the color maps of log$|L_{21}|$ as a function of the swept frequency and of the applied field for **k** ⊥ **n**. For **k** = 0.6 and 2.3 rad/μm, we observe the dominance of one spin-wave mode and at k= 1.2 rad/ μm with an increase in frequency of about 1 GHz for the dominant mode. We also observe the presence of a secondary spin-wave branch lower in amplitude and in frequency for **k** = 1.1 rad/μm, which goes beyond standard analysis and would require further investigation using for example Brillouin Light Scattering.

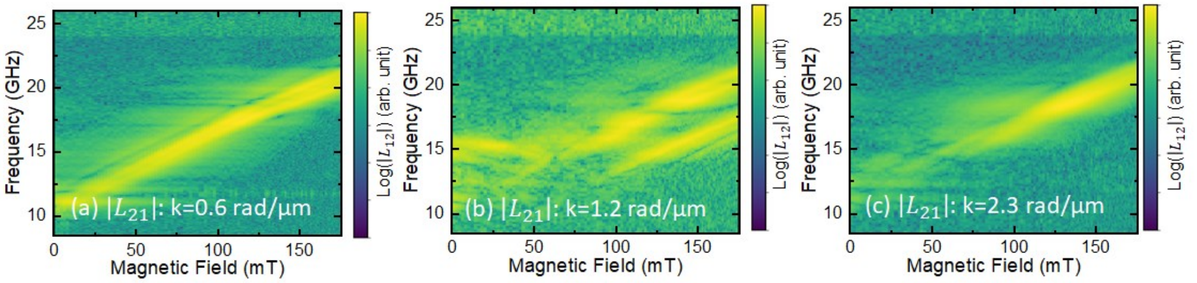

Figure S5: Frequency transmission spectra of the three designs for k ⊥ n: *(a) Color map of* $|\mathbf{L_{21}}|$ *for the design 1 having an excitation k at 0.6 rad/µm, (b) Color map of the absolute value of* L_{21} *for the Design 2 having an excitation k at 1.2 rad/µm, (c) Color map of the absolute value of* L_{21} *for the Design 3 having an excitation k at 2.3 rad/µm.*

In **Figure S6**, we then show the results for **k** // **n**, we observe a clear increase in frequency between k = 0.6 rad/μm and 2.3 rad/μm for the main spin-wave peak, corresponding to the bulk spin-wave mode. One should however notice that more secondary spin-wave peaks can be observed for k = 0.6 rad/μm, as the devices are larger and enable a more efficient detection. The frequency of the spin-wave mode with surface character has a smaller increase than expected for a surface mode (see **Suppl. Mat 6** for detailed experiments and **Suppl. Mat. 7** for detailed theory). Also, in this case, the device with k =1.2 rad/μm shows more pronounced secondary spin-wave peaks, which could indicate that the mode hybridization is maximum for this range of **k** vectors.

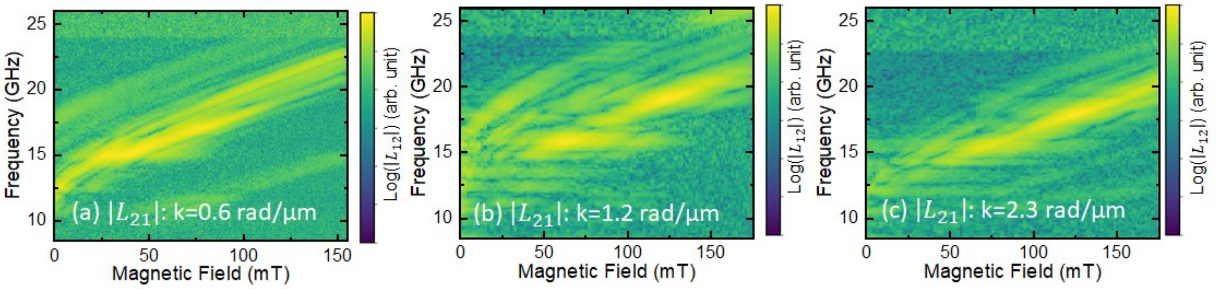

Figure S6: Transmission spectra of the three designs for H ⊥ k // n: *(a) Color map of* $|\mathbf{L_{21}}|$ *for the design 1 having an excitation k at 0.6 rad/µm, (b) Color map of the absolute value of* L_{21} *for the design 2 having an excitation k at 1.2 rad/µm, (c) Color map of the absolute value of* L_{21} *for the design 3 having an excitation k at 2.3 rad/µm.*

Section 4. Spin-wave non-reciprocity for k // n and k ⊥ n:

Non-reciprocity is a common feature observed for surface spin-waves in the case of ferromagnets(*10*). However, in antiferromagnets, non-reciprocal spin-waves have until now only been predicted for the spin-flopped phase of an easy-axis antiferromagnet (*7*, *8*). As shown in the main text, we observe non-reciprocity for some spin-waves modes for the case **k** // **n** which is in line with the theoretical predictions of Refs. (*7*, *8*).

Here after, we present some detailed data of the spin-wave transmission properties of L_{12} and L_{21} for positive and negative fields that complement the data from **Fig. 4** in the main text. In **Fig. S7**, we present L_{12} and L_{21} for positive and negative fields for **k**//**n** for the largest antennas (Design 1 with an edge to edge distance of 14 μm between antennas) in order to have the largest amplitude response, and on the spin-waves that arrive after more than 2 ns of travel time which are stable over the entire field range. We observe that L_{12} and L_{21} have a reverse behaviour for positive and negative fields, the signal being larger for the two non-reciprocal modes identified in the main for L_{12} for negative fields and for L_{21} positive fields. This observation further confirms the non-reciprocal behaviour of these antiferromagnetic spin-wave modes.

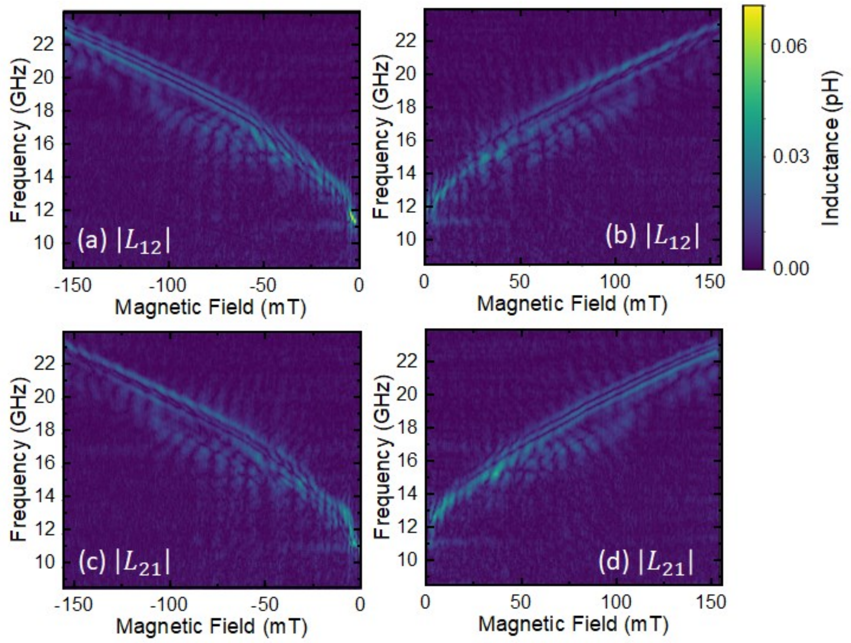

Figure S7: Transmission spectra in k // n with [2:50ns] gating: *(a) The* $|\boldsymbol{L_{12}}|$ *as a function of the frequency and of the negative magnetic field; for this case three peaks are observed. (b) The* $|\boldsymbol{L_{12}}|$ *as a function of frequency and positive magnetic field; for this only the highest mode of (a) is still observed clearly while the two lowest branches are hardly seen. In (c),(d) we repeat the same spectra of (a) and (b) but with* $|\boldsymbol{L_{21}}|$*, the peaks apparition and disappearance are reversed as expected.*

In **Fig. S8**, we then present for the same devices L_{12} and L_{21} for positive and negative fields for **k⊥n** in the same experimental conditions. In this case, we observe a slight difference for positive and negative fields at magnetic fields between 100-150 mT. However, we can associate it with the small hysteretic behavior in the sample and not to a potential non-reciprocity as we measure the same signals for L_{12} and L_{21}. This latter feature also indicates that our devices have nearly symmetric antennas and that our setup has well calibrated radiofrequency properties. The absence of non-reciprocity in this second configuration is also here in line with standard predictions for bulk antiferromagnetic spin-waves(*7, 8, 17, 54*). We also notice that the spin-wave amplitude is about 3-5 times smaller for **k⊥n** compared to **k**//**n**.

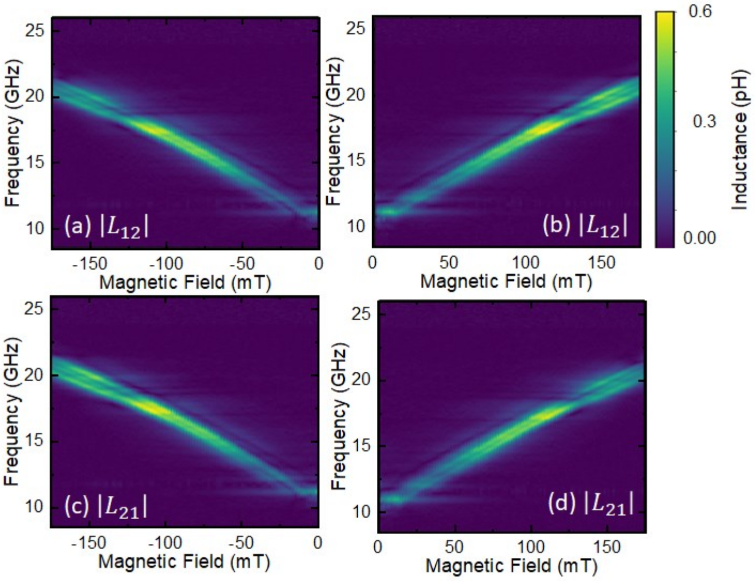

Figure S8: Transmission spectra for k⊥n without time gating *(a-b) The* $|\mathbf{L_{12}}|$ *as a function of frequency and the negative magnetic field. In (c-d), we repeat the same measurements for* $|\mathbf{L_{21}}|$ *and observe similar results.*

- **Example of non-reciprocity spectra for k = 2.3 rad/um:**

In **Fig. S9**, we show the frequency versus field maps sample design with **k** = 2.3 rad/µm for **k//n**. Here also we evidence a nonreciprocal behavior with larger amplitude at high field for one field direction**.** This evidences that the non-reciprocity is also present at larger **k**, in line with the theory developed in **Suppl. Mat S8**. One can also notice here that the non-reciprocity is also present for the main spin-wave branch, indicating the presence of mode hybridization between the bulk spin-wave mode and the surface modes which has been reported in ferromagnets (*46*) and therefore goes beyond the standard theory developed later.

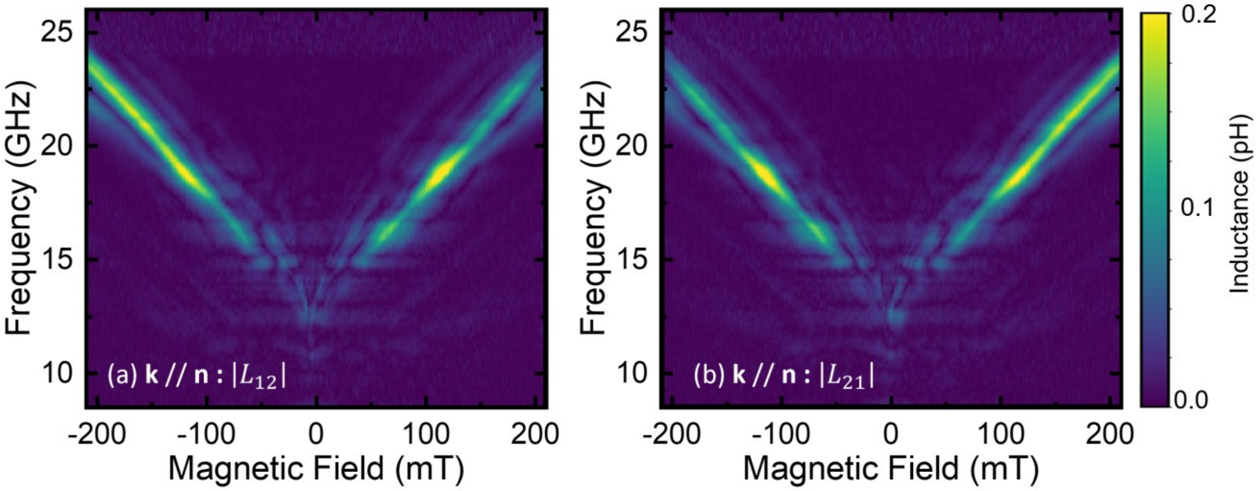

Figure S9: The transmission spectra (*$|\mathbf{L_{12}}|$ *and* $|\mathbf{L_{21}}|$*) for k⊥n (a-b) as a function of magnetic field for design 2 with k = 2.3 rad/um: *(a-b)* ***k⊥n****: Clear difference in intensity from negative to positive fields, dominance of spin-waves in negative fields for* $|\mathbf{L_{12}}|$ *(a), and in positive fields for* $|\mathbf{L_{21}}|$ *(b).*

Section 5. Magneto-static spin-wave in antiferromagnets

In antiferromagnets, in absence of dipole-dipole effects, spin waves are degenerate and can propagate only in the bulk with a standard quadratic dispersion in **k**. In presence of dipolar effects, the following modifications of spin wave modes are possible (*7, 17*). First, oscillations of the Néel vector induce nonzero magnetization leading to a lift of the degeneracy of the bulk spin-wave modes (see **Section 5a**). If this dynamic magnetization is oriented perpendicular to the sample surface, surface modes might also appear (see **Section 5b**). The origin of the surface mode in this case is the magnetostatic dipolar field which should vanish outside the film and penetrate into the sample until a finite length. In case of the canted antiferromagnets, additional contributions into the spectra can appear due to oscillations of the nonzero magnetic moments.

a. Bulk magnon spectra

Here we consider magnon spectra of an easy-plane canted antiferromagnet. The sample surface is parallel to the easy plane, the external magnetic field $\boldsymbol{H}$ is applied parallel to the easy plane, perpendicular to equilibrium orientation of the Néel vector $\boldsymbol{n_0}$, $\boldsymbol{H} \perp \boldsymbol{n_0}$.
In neglection of dipolar effects, magnon spectra split into two branches

$$\boldsymbol{f_1} = \sqrt{\boldsymbol{f_{10}^2} + \frac{\boldsymbol{c^2k^2}}{(\boldsymbol{2\pi})^2}}\,, \boldsymbol{f_2} = \sqrt{\boldsymbol{f_{20}^2} + \frac{\boldsymbol{c^2k^2}}{(\boldsymbol{2\pi})^2}} \qquad (Eq.\ S1)$$

with different frequency gaps, f_{10} and f_{20} and the same phase velocity c whose value does not depend on propagation direction. The lower branch with the frequency f_1 corresponds to in-plane oscillations of the Néel vector $\boldsymbol{n}$, while the upper branch with the frequency f_2 corresponds to out-of-plane oscillations of $\boldsymbol{n}$. In presence of the magnetic field(*41*)

$$\boldsymbol{f_{10}} = \left(\frac{\gamma}{2\pi}\right)\sqrt{\boldsymbol{H_{ex}H_{an-in}} + \boldsymbol{H}(\boldsymbol{H_{DMI}} + \boldsymbol{H})}, \boldsymbol{f_{20}} = \left(\frac{\gamma}{2\pi}\right)\sqrt{\boldsymbol{H_{ex}H_{an-out}} + \boldsymbol{H_{DMI}}(\boldsymbol{H_{DMI}} + \boldsymbol{H})} \qquad (Eq.\ S2)$$

where γ is gyromagnetic ratio, H_{ex} is exchange field that keeps sublattice magnetizations antiparallel, H_{DMI} is DMI field that induces canting of magnetic sublattices, H_{an-in} and H_{an-out} parametrize in-plane and out-of-plane anisotropy. Magnons are considered as excitations above an equilibrium state with the Néel vector $\boldsymbol{n_0}$ and nonzero magnetization $\boldsymbol{m}_0 = \frac{\boldsymbol{H_{DMI}\hat{z}\times n_0+H}}{H_{ex}}$, $\boldsymbol{m}_0 \perp \boldsymbol{n_0}$.
It should be noted that in contrast to compensated antiferromagnets, the orientation of the Néel vector can be reversed by the reversal of $\boldsymbol{H}$, due to coupling between $\boldsymbol{m}_0$ and $\boldsymbol{n_0}$ imposed by DMI. To include magnetostatic effects, we calculate magnon spectra by solving coupled equations for fluctuations of the Néel vector and the potential of dipole field ψ (*7, 19*). We first focus on the dipolar effects that modify magnon spectra compared to **Eqs. (S1-2)**. In canted antiferromagnets dipolar effects appear due to a nonzero magnetization that originates i) from canting of the magnetic

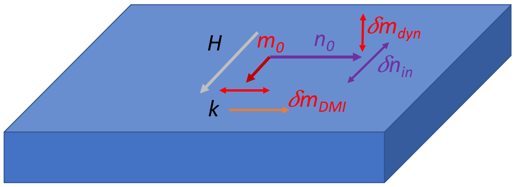

Figure S10. In-plane mode and dipolar effects. In-plane oscillations of the Néel vector, $\delta\boldsymbol{n}_{in}$, create an out-of-plane dynamic magnetization $\delta\boldsymbol{m}_{dyn}$. In addition, DMI-induced magnetization oscillates in plane (vector $\delta\boldsymbol{m}_{DMI}$). Magnons that propagate perpendicular to the magnetic field ($\boldsymbol{k} \perp \boldsymbol{H}, \boldsymbol{k}//\boldsymbol{n}_0$) create additional dipolar field due to oscillations of $\delta\boldsymbol{m}_{DMI}$. Dynamic magnetization $\delta\boldsymbol{m}_{dyn}$ creates magnetic charges at the sample surface and is responsible for formation of surface waves.

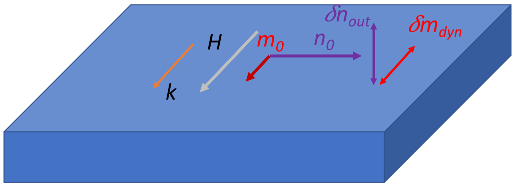

Figure S11. Out-of-plane mode and dipolar effects. Out-plane oscillations of the Néel vector, $\delta\boldsymbol{n}_{out}$, create an in-plane dynamic magnetization $\delta\boldsymbol{m}_{dyn}$. which creates a dipolar field for magnons propagating parallel to the magnetic field ($\boldsymbol{k}//\boldsymbol{H}, \boldsymbol{k} \perp \boldsymbol{n}_0$)

sublattices, $\boldsymbol{m}_{can} = \frac{H_{DMI}\hat{z}\times\boldsymbol{n}+\boldsymbol{H}}{H_{ex}}$ due to DMI field and external magnetic field $\boldsymbol{H}$; ii) from dynamics magnetization due to oscillations of the Néel vector, $\boldsymbol{m}_{dyn} = \boldsymbol{n} \times \dot{\boldsymbol{n}}/(\gamma H_{ex})$.

Based on the known results for ferromagnets(*11*), we anticipate the following dipolar effects in canted antiferromagnets (see **Figures S10 and S11**). Here we focus on the in-plane mode that we measure experimentally in the main text. One should notice that out-of-plane mode dynamic magnetization oscillates in-plane and contributes only into the bulk mode with $\boldsymbol{k} \perp \boldsymbol{n}_0$ $(\boldsymbol{k}//\boldsymbol{H})$. First, as mentioned in the main text, for $\boldsymbol{k}//\boldsymbol{n}$ $(\boldsymbol{k} \perp \boldsymbol{H})$, we thus observe a contribution of the dipolar field into the gap for the bulk modes as :

$$f_{\boldsymbol{k}||\boldsymbol{n}_0} = \sqrt{f_{10}^2 + \left(\frac{1}{2\pi}\right)^2 \left[\frac{4\pi M_s}{H_{ex}} \gamma^2 (H + H_{DMI})^2 + c^2 k^2\right]} \quad \text{(Eq. S3)}$$

In this case magnetostatic (dipolar) contribution in to the energy gap is proportional to oscillations of the canted magnetization (terms with DMI and magnetic field), but it is diminished (compared to ferromagnets) due to the small factor $\frac{4\pi M_s}{H_{ex}}$ (around 0.1 T).

In case of $\boldsymbol{k} \perp \boldsymbol{n}_0$ having nonzero component also in **z** direction, dipolar field can couple with the in-plane and out-of-plane modes and the resulting spin-wave mode has the following frequency:

$$f_{\boldsymbol{k}\perp\boldsymbol{n}_0}^2 = \frac{1}{2}\left(f_1^2 + f_2^2 + \frac{4\pi M_s}{H_{ex}}(f_1^2 \sin^2\theta + f_2^2 \cos^2\theta)\right) - \frac{1}{2}\sqrt{\left[f_1^2 + f_2^2 + \frac{4\pi M_s}{H_{ex}}(f_1^2 \sin^2\theta + f_2^2 \cos^2\theta)\right]^2 - 4\left(1 + \frac{4\pi M_s}{H_{ex}}\right) f_1^2 f_2^2} \quad \text{(Eq. S4)}$$

Where θ is the angle between **k** vector and z-axis. Dipolar fields thus contribute into the energy gap and also favor inhomogeneous distribution of the magnetization. This results in the softer dependence $f(\boldsymbol{k})$, and can lead to a negative group velocity at small $\boldsymbol{k}$ for non-zero $\mathbf{k_z}$ ($\theta\neq\pi/2$). At larger $\boldsymbol{k}$, dipolar fields are balanced by the exchange interactions that favour homogeneous ordering, and $f(\boldsymbol{k})$ dependence show always positive group velocity. Below we show in **Fig. S12** a representation of graph for non-zero k in the z direction.

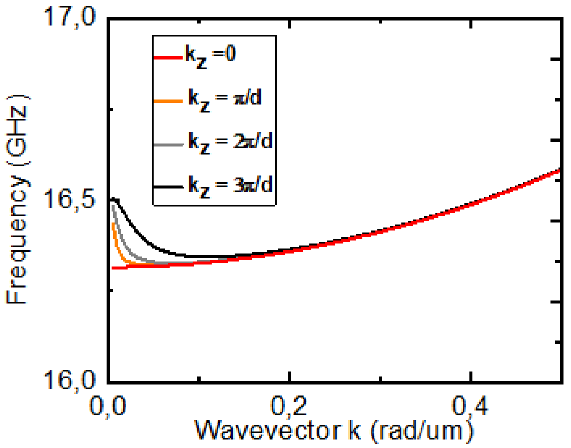

***Figure S12 Frequency dispersion of $\boldsymbol{k} \perp \boldsymbol{n}_0$ modes with non-zero $k_z = 0$**, indicating a non-uniform profile of the spin-wave mode within the bulk of the sample. The material parameters to compute these curves are the same as in the main text, see Table S2*

This could experimentally be observed due to the non-uniform radiofrequency magnetic field generated by our antenna over the sample thickness, resulting in the presence of secondary spin-wave peaks. However, considering the antenna design (**Suppl. Mat S1**), we do not expect these modes to dominate in the experiments.
In absence of **z**-component of the wave vector (θ≠π/2), the **Equation S5** for the bulk mode is then reduced to (as in the main text):

$$f_{\boldsymbol{k}\perp\boldsymbol{n}_0} = \left(1 + \frac{4\pi M_s}{H_{ex}}\right)\sqrt{f_{10}^2 + \left(\frac{ck}{2\pi}\right)^2} \text{ (Eq. S5)}$$

with positive dispersion. Due to smallness of $\xi = \frac{4\pi M_s}{H_{ex}} \ll 1$ this dispersion is close to f_1 at **k** = 0.

	**k ⊥ n**	**k // n**
H_{DMI} (T)	2 (blue) - 1.9 (brown)	1.8
Freq γH_{DMI} (GHz)	56 - 52	50
f_{10} (GHz)	10,3 (blue) – 12 (brown)	9.5 (lower red) – 1.5 (higher red)
$F2_0$ (GHz)	184	184
$\xi = 4\pi M_s/H_{ex}$	0.013	0.013
c (km/s)	24.5	24.5

Table S2 Material parameters obtained from fitting the experimental frequency dispersions for $\boldsymbol{k} \perp \boldsymbol{n_0}$ ***and*** $\boldsymbol{k}||\boldsymbol{n_0}$***, and as a function of k.***

Below we also show in **Fig. S13** the fits obtained from fitting the reflection spectra $|L_{11}|$ for **k** ⊥ **n** and **k** // **n,** corresponding to the insets of **Figure 1 (b-c).** One can see that for **k** // **n,** the $|L_{11}|$ parameter shows a resonance peak at lower frequency than for the transmission signal $|L_{21}|$ (shown in **Fig. 1** from the main manuscript) which could indicate that it is mainly sensitive to the uniform magnetization dynamics (k =0).

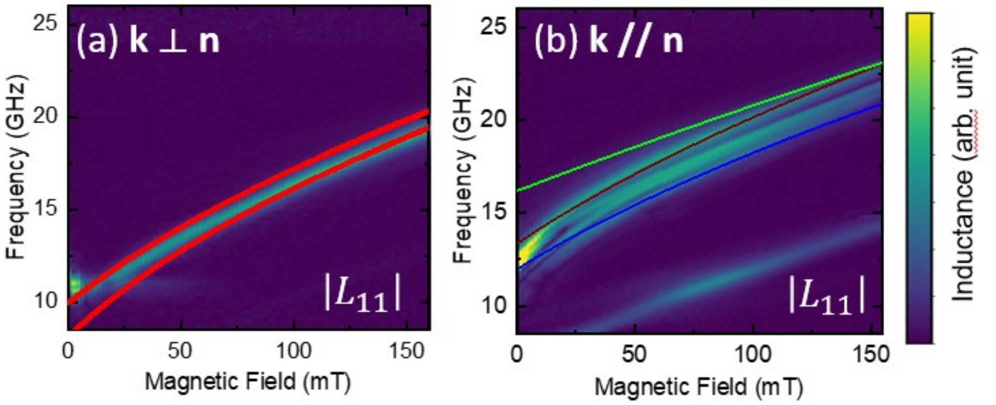

Figure S13: Reflection spectra of the L_{11} ***parameters for antenna with a k selectivity around 0.6 rad/µm. Lines correspond to the fits in the main text with the parameters from Table S2.***

In the limit of **k** vector within the center of the Brillouin zone, we determine the following group velocity for the bulk spin-wave modes:

$$v_g = \frac{c^2 k}{2\pi f(k;H)} \ \textbf{(Eq. S6)}$$

where the frequency $f(k;H)$ of the spin-wave is given by Eqs. 3-5 and depends on the magnetic field. For a fixed value of the **k** vector, $f(k;H)$ increases with the field and hence the group velocity decreases.

b. Surface spin-waves

The presence of dipolar field can result in appearance of the surface modes. For this case, one needs an out-of-plane component of the magnetization which in our case appears due to dynamic magnetization $\boldsymbol{n}\times\dot{\boldsymbol{n}}$. However, though oscillations of DMI-induced canted magnetization are always in-plane for this crystal orientation they also contribute into formation of the surface mode. Additionally, the stabilization of surface spin-waves requires a negative permeability within the bulk, in order to prevent penetration of oscillations of the magnetization into the sample bulk, which arises from the presence of a canted moment.

As such, surface in-plane modes are allowed, for any orientation of propagation vector within the plane. To model the system, we still follow the approach from Refs. (*7, 19*) and use magnetostatic boundary conditions at one of the sample surfaces, while assuming that the sample is thick enough and that the influence of the other surface can be neglected. We then obtain the following formula for the frequency of the surface mode for **k** // **n**:

$$f_{sur} = \frac{f_{10}^2+\left(\frac{ck}{2\pi}\right)^2}{\frac{\gamma(H+H_{DMI})}{\pi}} + \frac{\gamma}{4\pi}\left(1+\frac{4\pi M_s}{H_{ex}}\right)(H+H_{DMI}) \text{ (Eq. S7)}$$

Here z-component of vector $\boldsymbol{k}$ is imaginary.

In **Fig. S5,** we plot for the two following sets of parameters (fitted from experiments as in the main text, or theoretical ones with smaller $H_{DMI} = 1.3\ T$). Surface mode exists only in a certain range of the k vectors $k > k_{cr}$, at which spin-wave cannot propagate into the bulk. One can thus notice that surfaces modes are not supposed to be stable in the experimental accessible range of **k** vectors with H_{DMI} = 2 T, and above a critical $k_{cr} \approx 3$ rad/µm for a lower H_{DMI} and larger M_s/H_{ex}. This could indicate that these surface modes should be more easily detectable in Brillouin Light Scattering experiments. The discrepancy between the experiments and the theory could indicate that the surface of hematite have slightly different parameters than the bulk as reported in some articles(*55, 56*).

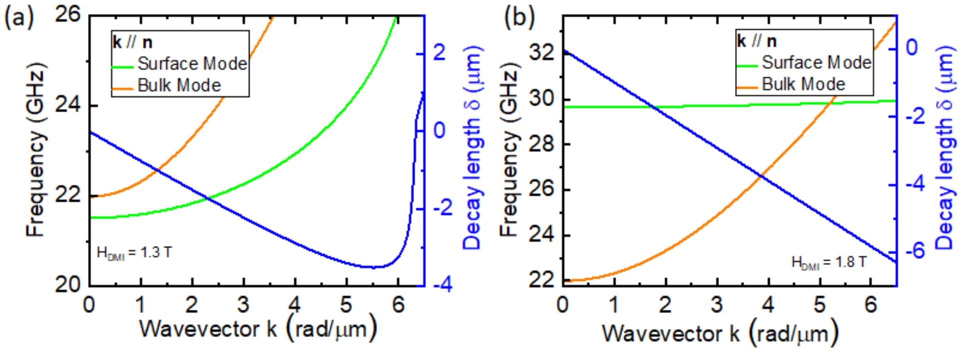

Figure S14 Frequency and decay length of the surface spin-waves modes for k // n*. (a) The* $\boldsymbol{H_{DMI}}$ *= 1.3 T and ratio* $\boldsymbol{M_s/H_{ex}}$*= 0.2 are fixed in this figure. Other parameters remain the same as in Table S2. (b) Case corresponding to the experimental parameters given in Table S2.*

These above conditions are related to the fact that surface modes are only stable when the permeability in the bulk of the material is negative, leading to the following condition on the decay length δ:

$$\delta = -k\frac{(1+\chi_{\{zx\}})}{1+\chi_{\{zz\}}} > 0 \text{ (Eq. S8)}$$

The diagonal component $\chi_{\{zz\}}$ corresponds to the out-of-plane dynamic magnetization $\boldsymbol{n}\times\dot{\boldsymbol{n}}$, it depends on the frequency of oscillations, anisotropy H_a, and M_s/H_{ex}. $\chi_{\{zx\}}$ corresponds to the out-of-plane oscillations of the in-plane nonzero magnetization and is proportional to H_{DMI} (and thus linked to the presence of a canted moment) and frequency f_{sur}, and also M_s/H_{ex}. $1+\chi_{\{zx\}}$and $1+$

χ_{zz} should have different signs. One should notice that if nondiagonal susceptibility is zero (i.e no canted moment), no localised solution is possible.
If DMI is extremely large and anisotropy is small, the frequency is large and the factors have the same sign. If DMI is zero, there is no canted magnetization and thus no coupling with the dynamic magnetization can occur. Here, due to the low magnetic anisotropy of hematite, the gap of the bulk spin-wave mode is particularly small so that one requires a small DMI field to lower the frequency of the surface mode (see **Eq. S5** for the surface mode) in order to fulfil this condition.

In the limit of k vector within the center of the Brillouin zone, we determine the following group velocity for the bulk spin-wave modes, we can also determine the group velocity of the surface spin-wave modes:

$$v_g = \frac{c^2 k}{2\pi f_D + \gamma H} \text{ (Eq. S9)}$$

With $f_D = \gamma H_{DMI}/2\pi$. One can notice that the surface spin-wave group velocities also decrease as a function of the applied magnetic field, in line with the behaviour of the slower spin-wave modes measured (blue dots) in **Fig. 3c** of the main manuscript.

Section 6. Angular dependency of the power absorbed in the antiferromagnet

The absorbed power in the magnetic material is given by $P_{abs} = \frac{\omega}{2}(\chi_\perp |h_\perp|^2 + \chi_{//}|h_{//}|^2)$. For a magnetic field H applied in the sample with an angle θ from the transducer axis, we thus get the two components of the rf-excitation field $h_\perp = h_x \cos\theta\, e_\perp + h_z e_z$ and $h_{//} = h_x \sin\theta\, e_{//}$ (see **Fig. S15**). We can then obtain (*57*):

$$P_{abs} = A\cos\theta^2 + B \text{ with } A = \frac{\mu_0\omega}{2}(\chi_\perp |h_x|^2 - \chi_{//}|h_x|^2) \text{ and } B = \frac{\mu_0\omega}{2}(\chi_\perp |h_z|^2 - \chi_{//}|h_x|^2)$$

The angular dependence of the inverse spin-Hall voltage shown in **Fig. 5** from the main is then proportional to the cross product of the absorbed power P_{abs} with the detection of the inverse spin-Hall effect (in $\sin\theta$).

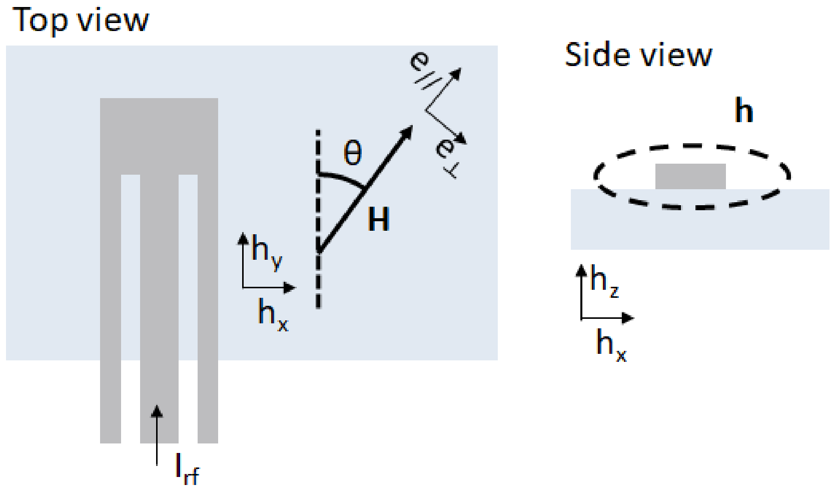

Figure S15 Sketch of the antenna with the rf excitation fields

Section 7. Magnetic characterization of hematite single crystals

We characterized the amplitude of the canted moment at room temperature in the purchased single crystals using a standard vibrating sample magnetometer (VSM). In **Fig. S16**, we performed measurements for a magnetic field applied in the sample plane, and extracted a total magnetic moment of about 25 memu for the canted moment of a single crystals with a size of 5 x 5 x 0.5 mm^3. This leads to a canted moment of about 2 emu/cm^3 in line with previous reports in single crystals(*58*, *59*). One can notice that a recent article on spin-wave transport in hematite (60) used single crystals with a value about 50% lower . This lower dipolar coupling could thus contribute to the absence of surface spin-wave reported in this recent study.

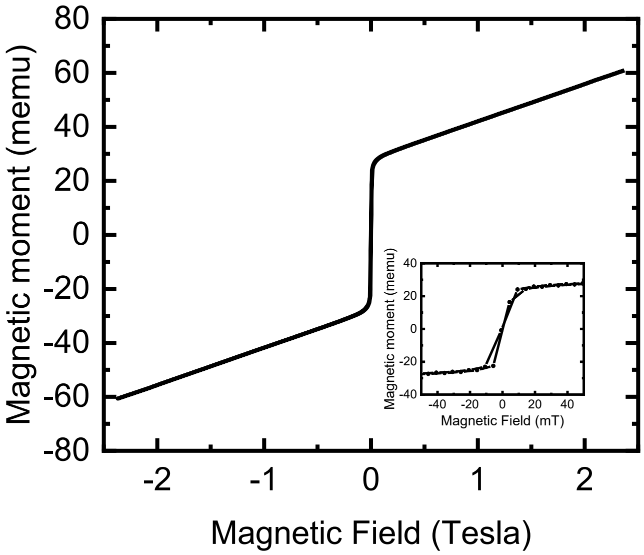

Figure S16 : Magnetic moment of the sample when applying a magnetic field within the sample plane (c-plane, easy-plane of hematite). Inset corresponds to a zoom at low magnetic fields to show the hysteresis loop associated with switching of the canted moment.

We also performed VSM for a magnetic field applied perpendicular to the sample plane to check the orientation of the small canted moment. In **Fig. S17**, we observe the absence of switching of the canted moment which is in line with the fact that the c-axis is a hard axis in the canted easy-plane phase of hematite. At an applied magnetic field of up to 2.5T, the measured net moment is approximately 30 memu, which remains considerably lower than the net moment (60 memu) that is observed when applying a magnetic field within the sample plane. This indicates that a magnetic field of 2.5T is too low to align the canted moment along the applied magnetic field. Therefore, one cannot investigate the possibility to stabilize surface spin-waves with an out-of-plane canted moment with this orientation of single crystals(7, 8, 54).

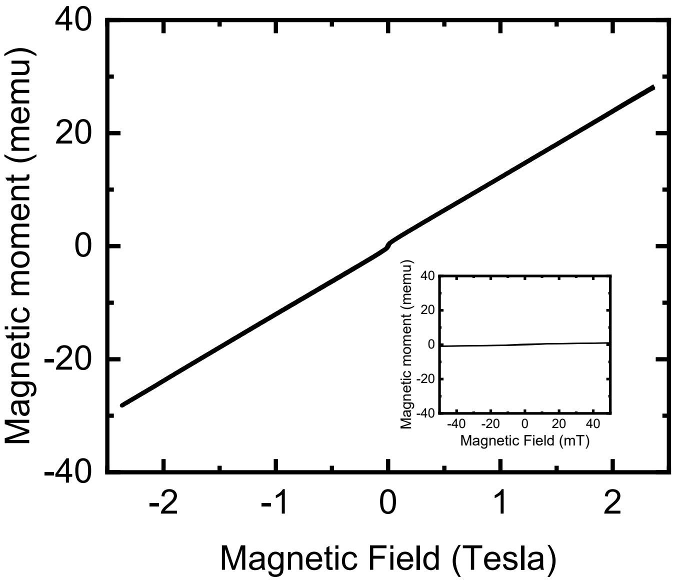

Figure S17: Magnetic moment of the sample when applying a magnetic field perpendicular the sample plane (c-plane, easy-plane of hematite). Inset corresponds to a zoom at low magnetic fields and show the absence of switching of the canted moment.

Section 8. Spin-wave spectroscopy for a magnetic field applied perpendicular to the sample plane

We also performed spin-wave spectroscopy measurements for a magnetic field applied perpendicular to the sample plane (c-plane). Given the large out-of-plane anisotropy, the spin-wave frequency is nearly constant as a function of the applied magnetic field as shown in **Fig. S18** and as expected from Ref. (*41*). The small increase of spin-wave frequency with fields can be associated with a small misalignment and the discontinuous signals at small magnetic fields (< 30 mT) with domain reorientation within the sample plane.

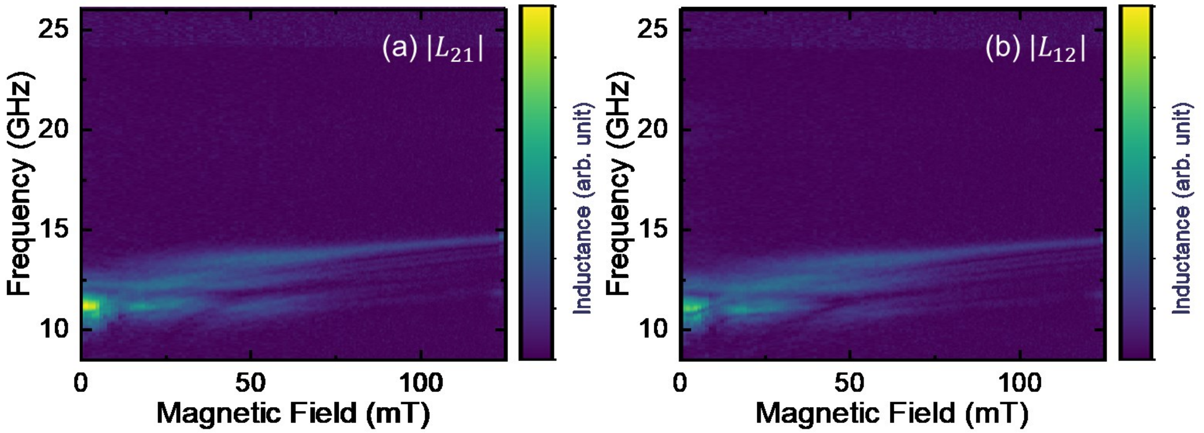

Figure S18: Spin wave transmission measurement showing the transmitted amplitude $|L_{21}|$ for a magnetic field H applied perpendicular to the sample plane (c-plane) at k ≈ 0.6 rad/µm.

REFERENCES AND NOTES

1. T. Kampfrath, A. Sell, G. Klatt, A. Pashkin, S. Mährlein, T. Dekorsy, M. Wolf, M. Fiebig, A. Leitenstorfer, R. Huber, Coherent terahertz control of antiferromagnetic spin waves. *Nat. Photonics* **5**, 31–34 (2011).

2. J. Li, C. B. Wilson, R. Cheng, M. Lohmann, M. Kavand, W. Yuan, M. Aldosary, N. Agladze, P. Wei, M. S. Sherwin, J. Shi, Spin current from sub-terahertz-generated antiferromagnetic magnons. *Nature* **578**, 70–74 (2020).

3. P. Vaidya, S. A. Morley, J. van Tol, Y. Liu, R. Cheng, A. Brataas, D. Lederman, E. del Barco, Subterahertz spin pumping from an insulating antiferromagnet. *Science* **368**, 160–165 (2020).

4. J. R. Hortensius, D. Afanasiev, M. Matthiesen, R. Leenders, R. Citro, A. V. Kimel, R. V. Mikhaylovskiy, B. A. Ivanov, A. D. Caviglia, Coherent spin-wave transport in an antiferromagnet. *Nat. Phys.* **17**, 1001–1006 (2021).

5. V. G. Bar'yakhtar, B. A. Ivanov, M. V. Chetkin, Dynamics of domain walls in weak ferromagnets. *Sov. Phys. Uspekhi* **28**, 563–588 (1985).

6. R. E. Camley, Long-wavelength surface spin waves on antiferromagnets. *Phys. Rev. Lett.* **45**, 283–286 (1980).

7. B. Lüthi, D. L. Mills, R. E. Camley, Surface spin waves in antiferromagnets. *Phys. Rev. B* **28**, 1475–1479 (1983).

8. B. Lüthi, R. Hock, Dipolar surface spin waves in antiferromagnets. *J. Magn. Magn. Mater.* **38**, 264–268 (1983).

9. R. L. Stamps, R. E. Camley, Bulk and surface spin waves in thin-film antiferromagnets. *J. Appl. Phys.* **56**, 3497–3502 (1984).

10. B. A. Kalinikos, A. N. Slavin, Theory of dipole-exchange spin wave spectrum for ferromagnetic films with mixed exchange boundary conditions. *J. Phys. C Solid State Phys.* **19**, 7013–7033 (1986).

11. J. R. Eshbach, R. W. Damon, Surface magnetostatic modes and surface spin waves. *Phys. Rev.* **118**, 1208–1210 (1960).

12. S. O. Demokritov, V. E. Demidov, O. Dzyapko, G. A. Melkov, A. A. Serga, B. Hillebrands, A. N. Slavin, Bose–Einstein condensation of quasi-equilibrium magnons at room temperature under pumping. *Nature* **443**, 430–433 (2006).

13. B. Divinskiy, H. Merbouche, V. E. Demidov, K. O. Nikolaev, L. Soumah, D. Gouéré, R. Lebrun, V. Cros, J. B. Youssef, P. Bortolotti, A. Anane, S. O. Demokritov, Evidence for spin current driven Bose-Einstein condensation of magnons. *Nat. Commun.* **12**, 6541 (2021).

14. T. Moriya, Anisotropic superexchange interaction and weak ferromagnetism. *Phys. Rev.* **120**, 91–98 (1960).

15. I. Dzyaloshinsky, A thermodynamic theory of “weak” ferromagnetism of antiferromagnetics. *J. Phys. Chem. Solid* **4**, 241–255 (1958).

16. W. Jantz, W. Wettling, Spin wave dispersion of $FeBO_3$ at small wavevectors. *Appl. Phys.* **15**, 399–407 (1978).

17. V. I. Ozhogin, Nonlinear dynamics of antiferromagnets with anisotropy of easy-plane type. *Sov. Phys. JETP* **21**, 874–881 (1965).

18. R. Orbach, Spin wave spectra for canted antiferromagnets and ferromagnets. *Phys. Rev.* **115**, 1189–1193 (1959).

19. V. V. Tarasenko, V. D. Kharitonov, Surface magnetostatic waves in uniaxial antiferromagnets. *Sov. Phys. JETP* **33**, (1971).

20. L. Šmejkal, J. Sinova, T. Jungwirth, Beyond conventional ferromagnetism and antiferromagnetism: A phase with nonrelativistic spin and crystal rotation symmetry. *Phys. Rev. X* **12**, 031042 (2022).

21. L. Šmejkal, J. Sinova, T. Jungwirth, Altermagnetism: A third magnetic class delimited by spin symmetry groups. arXiv:210505820 [cond-mat.mes-hall] (20 October 2021).

22. L. Šmejkal, A. Marmodoro, K.-H. Ahn, R. Gonzalez-Hernandez, I. Turek, S. Mankovsky, H. Ebert, S. W. D'Souza, O. Šipr, J. Sinova, T. Jungwirth, Chiral magnons in altermagnetic RuO2. arXiv:2211.13806 [cond-mat.mes-hall] (24 November 2022).

23. L. Šmejkal, J. Sinova, T. Jungwirth, Emerging research landscape of altermagnetism. *Phys. Rev. X* **12**, 040501 (2022).

24. M. Białek, A. Magrez, A. Murk, J.-Ph. Ansermet, Spin-wave resonances in bismuth orthoferrite at high temperatures. *Phys. Rev. B* **97**, 054410 (2018).

25. S. Das, A. Ross, X. X. Ma, S. Becker, C. Schmitt, F. van Duijn, E. F. Galindez-Ruales, F. Fuhrmann, M.-A. Syskaki, U. Ebels, V. Baltz, A.-L. Barra, H. Y. Chen, G. Jakob, S. X. Cao, J. Sinova, O. Gomonay, R. Lebrun, M. Kläui, Anisotropic long-range spin transport in canted antiferromagnetic orthoferrite $YFeO_3$. *Nat. Commun.* **13**, 6140 (2022).

26. G. F. Herrmann, Magnetic resonances and susceptibility in orthoferrites. *Phys. Rev.* **133**, A1334–A1344 (1964).

27. D. Treves, Magnetic studies of some orthoferrites. *Phys. Rev.* **125**, 1843–1853 (1962).

28. R. Lebrun, A. Ross, O. Gomonay, V. Baltz, U. Ebels, A.-L. Barra, A. Qaiumzadeh, A. Brataas, J. Sinova, M. Kläui, Long-distance spin-transport across the Morin phase transition up to room temperature in ultra-low damping single crystals of the antiferromagnet α-Fe_2O_3. *Nat. Commun.* **11**, 6332 (2020).

29. M. Białek, J. Zhang, H. Yu, J.-Ph. Ansermet, Antiferromagnetic resonance inα-Fe_2O_3 up to its Néel temperature. *Appl. Phys. Lett.* **121**, 032401 (2022).

30. R. Cheng, M. W. Daniels, J.-G. Zhu, D. Xiao, Antiferromagnetic spin wave field-effect transistor. *Sci. Rep.* **6**, 24223 (2016).

31. I. Proskurin, R. L. Stamps, A. S. Ovchinnikov, J. Kishine, Spin-wave chirality and its manifestations in antiferromagnets. *Phys. Rev. Lett.* **119**, 177202 (2017).

32. R. Cheng, D. Xiao, A. Brataas, Terahertz antiferromagnetic spin hall nano-oscillator. *Phys. Rev. Lett.* **116**, 207603 (2016).

33. I. Boventer, H. T. Simensen, A. Anane, M. Kläui, A. Brataas, R. Lebrun, Room-temperature antiferromagnetic resonance and inverse spin-hall voltage in canted antiferromagnets. *Phys. Rev. Lett.* **126**, 187201 (2021).

34. H. Wang, Y. Xiao, M. Guo, E. Lee-Wong, G. Q. Yan, R. Cheng, C. R. Du, Spin pumping of an easy-plane antiferromagnet enhanced by Dzyaloshinskii–Moriya interaction. *Phys. Rev. Lett.* **127**, 117202 (2021).

35. T. Devolder, G. Talmelli, S. M. Ngom, F. Ciubotaru, C. Adelmann, C. Chappert, Measuring the dispersion relations of spin wave bands using time-of-flight spectroscopy. *Phys. Rev. B* **103**, 214431 (2021).

36. A. V. Chumak, A. A. Serga, M. B. Jungfleisch, R. Neb, D. A. Bozhko, V. S. Tiberkevich, B. Hillebrands, Direct detection of magnon spin transport by the inverse spin Hall effect. *Appl. Phys. Lett.* **100**, 082405 (2012).

37. O. d'Allivy Kelly, A. Anane, R. Bernard, J. B. Youssef, C. Hahn, A. H. Molpeceres, C. Carrétéro, E. Jacquet, C. Deranlot, P. Bortolotti, R. Lebourgeois, J.-C. Mage, G. de Loubens, O. Klein, V. Cros, A. Fert, Inverse spin Hall effect in nanometer-thick yttrium iron garnet/Pt system. *Appl. Phys. Lett.* **103**, 082408 (2013).

38. A. H. Morrish, *Canted Antiferromagnetism: Hematite* (World Scientific, 1995); www.worldscientific.com/worldscibooks/10.1142/2518.

39. R. Lebrun, A. Ross, O. Gomonay, S. A. Bender, L. Baldrati, F. Kronast, A. Qaiumzadeh, J. Sinova, A. Brataas, R. A. Duine, M. Kläui, Anisotropies and magnetic phase transitions in insulating antiferromagnets determined by a Spin-Hall magnetoresistance probe. *Commun. Phys.* **2**, 50 (2019).

40. V. Vlaminck, M. Bailleul, Spin-wave transduction at the submicrometer scale: Experiment and modeling. *Phys. Rev. B* **81**, 014425 (2010).

41. H. J. Fink, Resonance line shapes of weak ferromagnets of the α−Fe_2O_3 and NiF_2 type. *Phys. Rev.* **133**, A1322 (1964).

42. D. E. Beeman, Magnetostatic modes in antiferromagnets and canted antiferromagnets. *J. Appl. Phys.* **37**, 1136–1137 (1966).

43. J. Han, P. Zhang, Z. Bi, Y. Fan, T. S. Safi, J. Xiang, J. Finley, L. Fu, R. Cheng, L. Liu, Birefringence-like spin transport via linearly polarized antiferromagnetic magnons. *Nat. Nanotechnol.* **15**, 563–568 (2020).

44. U. K. Bhaskar, G. Talmelli, F. Ciubotaru, C. Adelmann, T. Devolder, Backward volume vs Damon-Eshbach: A traveling spin wave spectroscopy comparison. *J. Appl. Phys.* **127**, 033902 (2020).

45. J. Cramer, U. Ritzmann, B.-W. Dong, S. Jaiswal, Z. Qiu, E. Saitoh, U. Nowak, Spin transport across antiferromagnets induced by the spin Seebeck effect. *J. Phys. Appl. Phys.* **51**, 144004 (2018).

46. I. V. Rojdestvenski, M. G. Cottam, A. N. Slavin, Dipole-exchange theory for Brillouin light scattering from hybridized spin waves in ferromagnetic thin films. *Phys. Rev. B* **48**, 12768–12777 (1993).

47. M. Jamali, J. H. Kwon, S.-M. Seo, K.-J. Lee, H. Yang, Spin wave nonreciprocity for logic device applications. *Sci. Rep.* **3**, 3160 (2013).

48. A. V. Chumak, P. Kabos, M. Wu, C. Abert, C. Adelmann, A. O. Adeyeye, J. Åkerman, F. G. Aliev, A. Anane, A. Awad, C. H. Back, A. Barman, G. E. W. Bauer, M. Becherer, E. N. Beginin, V. A. S. V. Bittencourt, Y. M. Blanter, P. Bortolotti, I. Boventer, D. A. Bozhko, S. A. Bunyaev, J. J. Carmiggelt, R. R. Cheenikundil, F. Ciubotaru, S. Cotofana, G. Csaba, O. V. Dobrovolskiy, C. Dubs, M. Elyasi, K. G. Fripp, H. Fulara, I. A. Golovchanskiy, C. Gonzalez-Ballestero, P. Graczyk, D. Grundler, P. Gruszecki, G. Gubbiotti, K. Guslienko, A. Haldar, S. Hamdioui, R. Hertel, B. Hillebrands, T. Hioki, A. Houshang, C.-M. Hu, H. Huebl, M. Huth, E. Iacocca, M. B. Jungfleisch, G. N. Kakazei, A. Khitun, R. Khymyn, T. Kikkawa, M. Kläui, O. Klein, J. W. Kłos, S. Knauer, S. Koraltan, M. Kostylev, M. Krawczyk, I. N. Krivorotov, V. V. Kruglyak, D. Lachance-Quirion, S. Ladak, R. Lebrun, Y. Li, M. Lindner, R. Macêdo, S. Mayr, G. A. Melkov, S. Mieszczak, Y.

Nakamura, H. T. Nembach, A. A. Nikitin, S. A. Nikitov, V. Novosad, J. A. Otálora, Y. Otani, A. Papp, B. Pigeau, P. Pirro, W. Porod, F. Porrati, H. Qin, B. Rana, T. Reimann, F. Riente, O. Romero-Isart, A. Ross, A. V. Sadovnikov, A. R. Safin, E. Saitoh, G. Schmidt, H. Schultheiss, K. Schultheiss, A. A. Serga, S. Sharma, J. M. Shaw, D. Suess, O. Surzhenko, K. Szulc, T. Taniguchi, M. Urbánek, K. Usami, A. B. Ustinov, T. van der Sar, S. van Dijken, V. I. Vasyuchka, R. Verba, S. V. Kusminskiy, Q. Wang, M. Weides, M. Weiler, S. Wintz, S. P. Wolski, X. Zhang, Advances in magnetics roadmap on spin-wave computing. *IEEE Trans. Magn.* **58**, 1–72 (2022).

49. J. Fischer, M. Althammer, N. Vlietstra, H. Huebl, S. T. B. Goennenwein, R. Gross, S. Geprägs, M. Opel, Large spin hall magnetoresistance in antiferromagnetic α−Fe_2O_3/Pt heterostructures. *Phys. Rev. Appl.* **13**, 014019 (2020).

50. M. Althammer, S. Meyer, H. Nakayama, M. Schreier, S. Altmannshofer, M. Weiler, H. Huebl, S. Geprägs, M. Opel, R. Gross, D. Meier, C. Klewe, T. Kuschel, J.-M. Schmalhorst, G. Reiss, L. Shen, A. Gupta, Y.-T. Chen, G. E. W. Bauer, E. Saitoh, S. T. B. Goennenwein, Quantitative study of the spin Hall magnetoresistance in ferromagnetic insulator/normal metal hybrids. *Phys. Rev. B* **87**, 224401 (2013).

51. H. Wang, R. Yuan, Y. Zhou, Y. Zhang, J. Chen, S. Liu, H. Jia, D. Yu, J.-P. Ansermet, C. Song, H. Yu, Long-distance propagation of high-velocity antiferromagnetic spin waves. arXiv:2211.10989 [cond-mat.mtrl-sci] (20 November 2022).

52. M. Hamdi, F. Posva, D. Grundler, Spin wave dispersion of ultra-low damping hematite (α-Fe_2O_3) at GHz frequencies. arXiv:2212.11887 [cond-mat.mtrl-sci] (23 December 2022).

53. Rohde & Schwarz, Time domain analysis with VNA; www.rohde-schwarz.com/fr/produits/test-et-mesure/options-pour-analyseurs-de-reseaux/time-domain-analysis_333648.html.

54. V. Veerakumar, R. E. Camley, Magnetostatic bulk and surface spin-wave focusing in antiferromagnetic thin films. *Phys. Rev. B* **81**, 174432 (2010).

55. D. S. Ellis, E. Weschke, A. Kay, D. A. Grave, K. D. Malviya, H. Mor, F. M. F. de Groot, H. Dotan, A. Rothschild, Magnetic states at the surface of $\alpha-Fe_2O_3$ thin films doped with Ti, Zn, or Sn. *Phys. Rev. B* **96**, 094426 (2017).

56. G. S. Krinchik, V. E. Zubov, Surface magnetism of hematite. *Sov. Phys. JEPT* **42***,* 359–366 (1975).

57. O. D'Allivy Kelly, "Détection et excitation d'ondes de spin dans des microstructures de couches ultraminces $Y_3Fe_5O_{12}$/métal à fort couplage spin orbite," thesis, Université Paris Saclay (2015).

58. A. H. Morrish, *Canted Antiferromagnetism: Hematite* (World Scientific, 1995).

59. R. Lebrun, A. Ross, S. A. Bender, A. Qaiumzadeh, L. Baldrati, J. Cramer, A. Brataas, R. A. Duine, M. Kläui, Tunable long-distance spin transport in a crystalline antiferromagnetic iron oxide. *Nature* **561**, 222–225 (2018).

60. H. Wang, R. Yuan, Y. Zhou, Y. Zhang, J. Chen, S. Liu, H. Jia, D. Yu, J.-P. Ansermet, C. Song, H. Yu, Long-distance coherent propagation of high-velocity antiferromagnetic spin waves. *Phys. Rev. Lett.* **130**, 096701 (2023).
